# North Sea spring bloom-associated *Gammaproteobacteria* fill diverse heterotrophic niches

**DOI:** 10.1186/s40793-021-00385-y

**Published:** 2021-08-17

**Authors:** Ben Francis, Tim Urich, Annett Mikolasch, Hanno Teeling, Rudolf Amann

**Affiliations:** 1 Max Planck Institute for Marine Microbiology, Bremen, Germany; 2 Institute for Microbiology, University of Greifswald, Greifswald, Germany

**Keywords:** Metagenome assembled genome, Metaproteome, Polysaccharide utilisation locus, Methylotrophy

## Abstract

**Background:**

The planktonic bacterial community associated with spring phytoplankton blooms in the North Sea is responsible for a large amount of carbon turnover in an environment characterised by high primary productivity. Individual clades belonging to the *Gammaproteobacteria* have shown similar population dynamics to *Bacteroidetes species*, and are thus assumed to fill competing ecological niches. Previous studies have generated large numbers of metagenome assembled genomes and metaproteomes from these environments, which can be readily mined to identify populations performing potentially important ecosystem functions. In this study we attempt to catalogue these spring bloom-associated *Gammaproteobacteria*, which have thus far attracted less attention than sympatric *Alphaproteobacteria* and *Bacteroidetes*.

**Methods:**

We annotated 120 non-redundant species-representative gammaproteobacterial metagenome assembled genomes from spring bloom sampling campaigns covering the four years 2010–2012 and 2016 using a combination of Prokka and PfamScan, with further confirmation via BLAST against NCBI-NR. We also matched these gene annotations to 20 previously published metaproteomes covering those sampling periods plus the spring of 2009.

**Results:**

Metagenome assembled genomes with clear capacity for polysaccharide degradation via dedicated clusters of carbohydrate active enzymes were among the most abundant during blooms. Many genomes lacked gene clusters with clearly identifiable predicted polysaccharide substrates, although abundantly expressed loci for the uptake of large molecules were identified in metaproteomes. While the larger biopolymers, which are the most abundant sources of reduced carbon following algal blooms, are likely the main energy source, some gammaproteobacterial clades were clearly specialised for smaller organic compounds. Their substrates range from amino acids, monosaccharides, and DMSP, to the less expected, such as terpenoids, and aromatics and biphenyls, as well as many ‘unknowns’. In particular we uncover a much greater breadth of apparent methylotrophic capability than heretofore identified, present in several order level clades without cultivated representatives.

**Conclusions:**

Large numbers of metagenome assembled genomes are today publicly available, containing a wealth of readily accessible information. Here we identified a variety of predicted metabolisms of interest, which include diverse potential heterotrophic niches of spring bloom-associated *Gammaproteobacteria*. Features such as those identified here could well be fertile ground for future experimental studies.

**Supplementary Information:**

The online version contains supplementary material available at 10.1186/s40793-021-00385-y.

## Background

Phytoplankton blooms represent massive perturbations in the marine surface water ecosystems in which they occur. Characterised by rapid rises and falls in algal populations, they produce large quantities of fixed organic carbon that is quickly transformed to dissolved and particulate organic matter (e.g. [1–4]). Three major clades of heterotrophic bacteria, namely the *Alpha-* and *Gammaproteobacteria* and the *Bacteroidetes*, have been found in many studies to grow in response to marine phytoplankton blooms [5–10]. The molecular complexity of algal organic matter is high [11–15], in theory necessitating the presence of multiple bacterial clades that can degrade distinct substrate spectra.

While the *Alpha*- and *Gammaproteobacteria* and *Bacteroidetes* each account for similar proportions of the overall bacterial community during blooms in terms of inferred cell numbers, it is the *Alphaproteobacteria* and *Bacteroidetes* that have attracted greater attention to date (e.g. [16–18]). In this study, we thus turn our attention to the *Gammaproteobacteria*, which are a significant part of both pre-bloom and bloom communities, and additionally have been found to directly respond to the phytoplankton growth and decline in a similar fashion to the *Bacteroidetes* [7–9].

The concept of ‘division of labour’ between the *Alpha-* and *Gammaproteobacteria* and *Bacteroidetes* has focused on the molecular weight of the algal-derived organic substrates consumed. *Bacteroidetes* are generally considered specialists for high molecular weight (HMW) food sources such as protein and polysaccharide [19–23]. Meanwhile, the expectation for the *Alphaproteobacteria* is that they typically prefer smaller organic molecules such as sugar monomers, amino acids, and diverse others such as dimethylsulfoniopropionate (DMSP), glycolate, amines and methylamines, urea, and phosphonates (e.g. [18, 24–26]). As a means of uptake the two groups have thus divergent requirements, with the *Alphaproteobacteria* typically employing small molecule transporters of the major facilitator superfamily (MFS), Tripartite ATP-independent periplasmic transporter (TRAP), and ATP-Binding Cassette (ABC) families, while the *Bacteroidetes* employ large repertoires of energy dependent TonB-dependent transporters (TBDTs) for large molecule requisition into the periplasmic space prior to downstream processing. The *Gammaproteobacteria* similarly make substantial use of TBDTs (e.g. [9, 27–29]), and it is expected to be for much the same reason as the *Bacteroidetes*, namely the ‘selfish’ [30] sequestration of substrate in order to maximise energy yield from these oligo- or polymers. Unlike in the *Bacteroidetes* however, this is not expected to be a universal strategy, with an overlap also expected between lifestyles of a number of gammaproteobacterial species and *Alphaproteobacteria*. Examples include the consumption of DMSP and other small organic molecules (widely reported in both clades) (e.g. [18, 31, 32]), putative associations with eukaryotes, and a related role for de novo vitamin synthesis by the bacteria [32–34].

Our aim with this study is to better understand the diversity of *Gammaproteobacteria* in temperate marine surface waters, and generate hypotheses about these organisms that may be further tested in future studies. We posit that in spite of the inevitable difficulties in annotating gene functions, publicly available metagenomic datasets can be a valuable tool for identifying potential organisms, metabolisms, and ecological trends of interest. And even a straightforward perusal of these annotations can generate worthwhile knowledge.

In this study specifically, we take the concept of ‘division of labour’, and attempt to identify heterotrophic specialties among the different gammaproteobacterial clades based on investigation of metagenome assembled genomes (MAGs), and corresponding metaproteomic data. These data derive from spring phytoplankton bloom sampling campaigns conducted over multiple years in the southern North Sea. These blooms are typically dominated by centric diatoms, with smaller contributions of *Phaeocystis* spp. and silicoflagellates [8]. Among the *Gammaproteobacteria*, the largest group we expect to find recurrently at this site are the bloom-responsive putative consumers of HMW organic matter, i.e. protein and polysaccharide, and derived oligopeptides and oligosaccharides. We also expect bloom-responsive consumers of low molecular weight organic matter (LMW), i.e. monosaccharides and amino acids, and likely disaccharides and di- and tripeptides, as well as some other abundant small molecules such as DMSP, and also the well documented metabolism of single carbon compounds by methylotrophs. Inevitably overlap in substrate consumption between the groups will happen, but the general pattern should be robust. With detailed study of gene annotations, however, we find practically every genome has a story to tell, that the diversity of predicted heterotrophic lifestyles among the *Gammaproteobacteria* in these environments is substantial, and that it spans a wider variety of potential organic substrates than seen among the bloom-responsive *Bacteroidetes*.

## Results and discussion

As is necessary for a compendium of data covering many genomes from a single environment, we present here in the main text what we consider the most interesting highlights from the MAGs, with more granular detail for each individual clade presented in the Additional file 1. The metagenomic data itself derives from a total of 47 surface seawater samples collected during spring blooms at Helgoland island in the North Sea in the four years 2010–2012 and 2016 [8, 17]. Samples were collected on 0.2 µm pore-size filters after pre-filtration through 10 µm and 3 µm filters, and thus represent the ‘free-living’ fraction of the bacterioplankton. Metaproteomes were extracted from equivalent samples, from 20 dates over the years 2009–2012, and 2016 [8, 9, 35]. MAGs were produced from the assemblies of each of the metagenomic datasets via automated binning followed by manual curation and dereplication.

### MAG quality

The North Sea spring bloom MAGs we used have been previously published [17], and were selected on the basis of having > 50% completeness and < 5% redundancy or contamination according to at least one of anvi’o [36] or CheckM [37]. Since the MAGs were manually refined, the overall level of contamination should be low. Many of the MAGs are more than 80 and 90% complete, however we included the less complete MAGs in this study because they still provide meaningful biological information. In general the less complete MAGs belong to clades that have more complete representatives from other species. Detail on completeness and contamination can be found in Additional file 2: Table S1.

### Phylogenetic diversity and clade abundance

From the complete collection of MAGs, we identified 120 approximate species-like clusters belonging to the class *Gammaproteobacteria* (henceforth ‘species’), and thus a single representative MAG for each. This is approximately 30% fewer than the equivalent number for *Bacteroidetes* (175), and approximately 20% more than for *Alphaproteobacteria* (98) (data not shown).

**Fig. 1:**
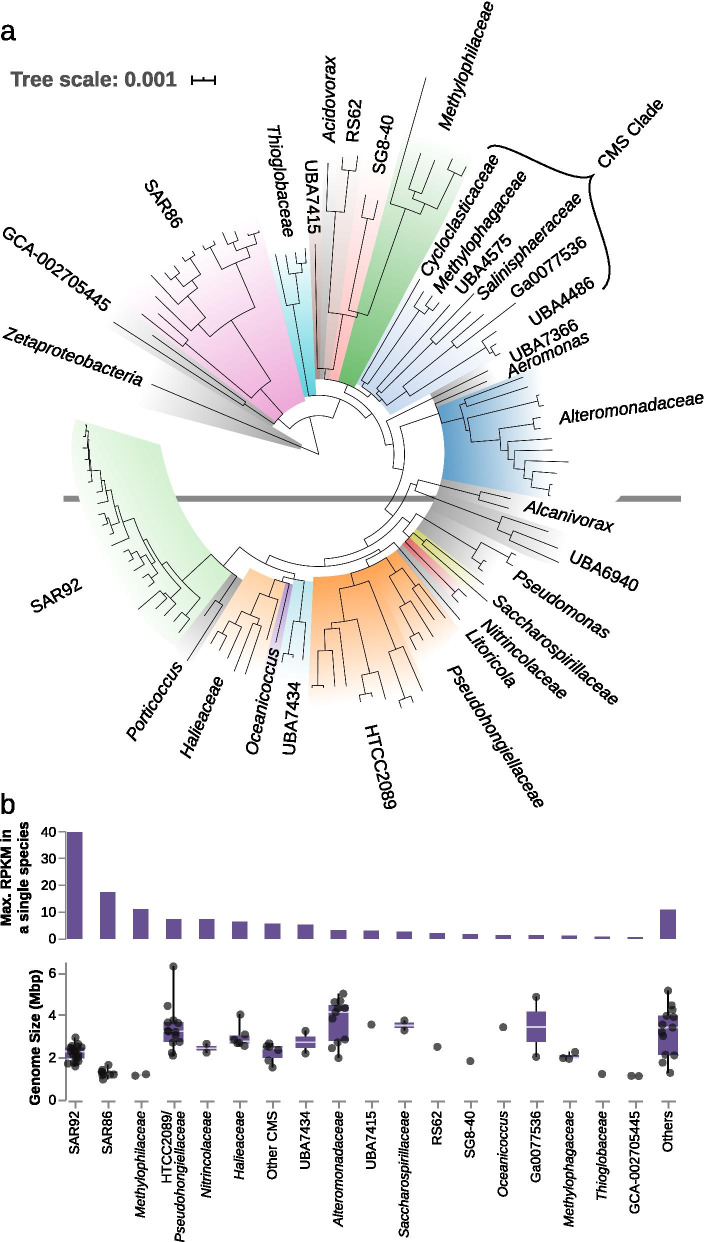
**a** Phylogenomic reconstruction of North Sea spring bloom associated gammaproteobacterial MAGs using GTDB-tk. **b** Distribution of maximum abundances and genome sizes for the major clades. Only genomes more than 80% complete are included. Values in upper part are the single highest abundance in reads per kilobase pair per million (RPKM) for any genome in that group in any individual sample. Points in lower part represent individual genomes

Genome based phylogeny inferred with GTDB-tk [38] indicates that four major radiations predominate (Fig. 1a). Namely, these are the genus-level SAR92 clade (22 species), the HTCC2089/*Pseudohongiellaceae* clade (15 species), the order-level SAR86 clade (14 species), and the *Alteromonadaceae* (12 species). Smaller radiations are also evident in the *Halieaceae* (6 species), *Methylophilaceae* (5 species), and *Thioglobaceae* (4 species). One final radiation of note is that of the clades *Cycloclasticaceae*, *Methylophagaceae*, *Salinisphaeraceae*, UBA4575, UBA4486, and Ga0077536 (Fig. 1a). The twelve species in these six families belong to six different orders making up a single monophyletic group. For convenience, we refer to this group as the CMS clade, for the three named families it contains.

16S rRNA gene based phylogenetic reconstruction produced a similar taxonomic view, although with slightly varying tree topology, and tentative assignments of certain individual species to specific genera. For further detail see the Additional file 1: subsection 16S rRNA gene based phylogenetic reconstruction and Additional file 1: Fig. S1.

The eight clades mentioned above constitute the bulk of the species diversity and are also among the most abundant in both metagenomic data (Fig. 1b), and in previously reported cell count data [8, 9], implying they comprise the majority of the *Gammaproteobacteria* biomass during the sampling periods. The clearest pattern to emerge from the read recruitment data is the distinction between those that strongly increase in relative abundance during blooms, and those groups that do not (analogous to a bloom community versus a pre-bloom/post-bloom community). In general, the most species rich groups tended to belong to the former category, while the less rich groups (e.g. the *Thioglobaceae* and several of the CMS clade) belonged to the latter. Further detail on individual clade abundances is included in the Additional file 1.

## Large-biopolymer degraders

**Fig. 2:**
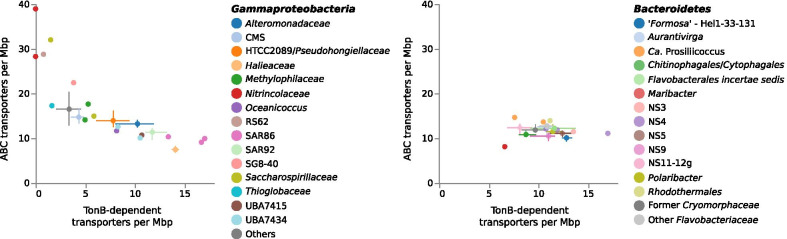
Numbers of annotated TonB-dependent transporters versus ABC transporters per megabase pair of genome. For clades with many representative genomes, mean values are plotted with lines indicating lower and upper quartiles. Only genomes estimated to be more than 90% complete were included

This collection of clades contains the most species, covering 74 of the 120. By virtue of their diversity and abundance (Fig. 1), the SAR92 and SAR86 clades that make up a major part of this category are likely the predominant gammaproteobacterial degraders of algal organic matter in this system. The other clades included in this group are the *Alteromonadaceae*, HTCC2089/*Pseudohongiellaceae*, the *Halieaceae*, and the UBA7434 clade. There are also two singleton genomes in this category, one in the genus *Oceanicoccus* (sister to the *Halieaceae*), and the other in the family UBA7415. The number of annotations of the variously annotated TonB-dependent transporters, including vitamin B12 transporter BtuB, colicin and pesticin receptors, ferrichrome-iron receptor, ferripyoverdine and ferrienterobactin receptors in these clades range from 5.5 to 20.8 per megabase pair (Fig. 2, left panel). Very similar numbers, but with lower variance, are seen among the *Bacteroidetes* (Fig. 2, right panel). These transporters are generally expected to be responsible for uptake of larger biopolymers such as oligopeptides and oligosaccharides, and are the most abundant protein category in the metaproteomic data from the Helgoland spring blooms [35].

### Expected functional diversity

**Fig. 3:**
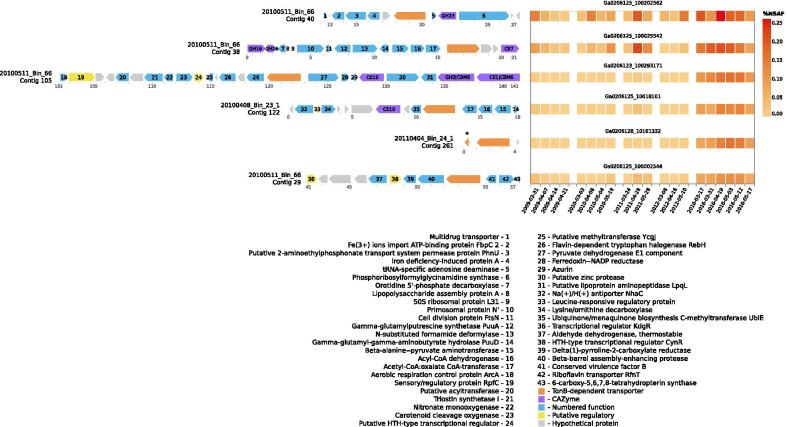
Six most abundant TonB-dependent transporters in metaproteomic data belonging to the SAR92 species with representative genome 20110523_Bin_31_1 (heatmap, right), with their genomic contexts (arrow plots, left). Metaproteome data is presented as normalised spectral abundance factor (%NSAF), which indicates abundance of a protein as a percentage of all protein in a sample. Gene arrangements are taken from several genomes belonging to this species, based on length of contig on which the gene was assembled. The fifth most abundant was present in only two genomes, on very short contigs. The asterisk (*) indicates which TBDT is represented in the metaproteome data. The abundance pattern in the metaproteome suggests that these contigs were not mistakenly assigned to this species, however. Numbers below arrows indicate position on contig in kilobase pairs

Prediction of transported substrates for TBDTs from genomes can be challenging unless proximity of CAZymes provides a clear signal of oligo- or polysaccharide transport. To illustrate this, Fig. 3 shows the six most abundant TonB-dependent transporters in metaproteomic data belonging to the most consistently abundant SAR92 species across the blooms studied, represented by the MAG *20110523_Bin_31_1*. The locus around the most abundant TBDT features two annotations for an Fe^3+^ ion binding protein and an iron deficiency-induced protein. These suggest a potential for “classical” iron-siderophore uptake by this transporter. The next two are co-located with CAZymes, while the fourth has 92% amino acid identity to the second, along with four other homologous genes. The final two TonB-dependent transporters in Fig. 3 do not lend themselves easily to interpretation, as is the case for most of the TBDTs present in the genomes studied.

However, for polysaccharides at least, we can make predictions regarding transported substrates. Indeed polysaccharide consumption falls into the category of ‘expected’ diversity present among spring bloom-associated *Gammaproteobacteria* (e.g. [9, 39]). The major polysaccharide substrates expected to be consumed by heterotrophic bacteria in these systems have been investigated in the *Bacteroidetes* [17], with the identification of polysaccharide utilisation loci (PULs) targeting the beta-glucan laminarin, alpha-glucans such as starch, glycogen, or pullulan, xylans, mannans, and alginates. The PUL-like structures present in the gammaproteobacterial MAGs lack the SusCD transporter tandem typical of *Bacteroidetes*, instead having other TonB-dependent transporters performing a similar role, as can be seen in the second and third loci in Fig. 3. To broaden coverage of functions, however, we did not restrict the overall analysis, and searched also for CAZyme clusters without co-located TonB-dependent transporters.

The overall diversity of the PULs in the *Gammaproteobacteria* MAGs is lower than in the *Bacteroidetes.* Automated prediction of PULs produced an initial total of 317 PULs in *Bacteroidetes* MAGs, versus 48 among *Gammaproteobacteria*. Taking only MAGs that had at least one PUL, the average number per MAG was 2.7 for the *Bacteroidetes*, and 1.5 for *Gammaproteobacteria.* Predicted laminarin degrading gene clusters are present in species of five of the six main clades in this section, plus each of the two singletons; those without are the UBA7434. These gene clusters contain various combinations of the glycoside hydrolase families GH3, GH16, GH17, GH30, and occasionally GH5 and GH81, and have been shown to be active in laminarin degradation in *Bacteroidetes* [40, 41]. Possible examples of these are the second and third loci in Fig. 3, however both GH3 and GH16 are large CAZyme families with many described activities. The presence of the carbohydrate-binding module CBM6 domains in the third locus in Fig. 3 would also point to activity on beta-glucans [42], however.

While predicted laminarin degrading gene clusters have been found to be ubiquitous in the spring-bloom *Bacteroidetes* [17, 43], in the *Gammaproteobacteria* they are restricted to presence in just six of the 22 SAR92 species, four each of the 14 SAR86 and 12 *Alteromonadaceae*, two of the 6 *Halieaceae* and one of the 15 HTCC2089/*Pseudohongiellaceae*. Numbers are smaller still for other predicted substrates, with alpha-glucan targeting clusters (containing GH13, GH57, GH77, and in some cases GH31 family genes) present in eight of the *Alteromonadaceae*, four SAR92, and one of the HTCC2089/*Pseudohongiellaceae.* Alginate degrading clusters (polysaccharide lyase families PL6, PL7, and PL17) are present in three species each from the *Alteromonadaceae* and SAR92. Finally among the five main substrates there are putative xylan targeting gene clusters (including combinations of GH10, GH11, GH43 family genes) present in three *Alteromonadaceae* (without sulfatases) and one of the three species of the UBA7434 (with sulfatases), while two *Alteromonadaceae* species have alpha-mannan targeting GH92 clusters, and one a cluster containing GH130, likely targeting a beta-mannan.

The glycan classes predicted for the *Gammaproteobacteria* are the same as those found among bloom-responsive *Bacteroidetes*. This gives further weight to the idea that these are the most important polysaccharides released by the phytoplankton during these events. We expect the small genome sizes of the SAR92, UBA7434, and especially the SAR86 (Fig. 1b) allow them to compete with the *Bacteroidetes* that are abundant at these times, which also have smaller genomes (in the range 1.5–2.5 Mbp [17]). In contrast the *Alteromonadaceae* genomes are much larger (3–5 Mbp), and less abundant in our metagenomic datasets (Fig. 1b; Additional file 1: section *Alteromonadaceae*). 16S rRNA gene amplicon data indicates that these species may well be more abundant in larger size fractions (see Additional file 1: Fig. S7). Their diversity and large genome size would be consistent with heterogeneous carbohydrate defined niches associated with particles and their degradation, in contrast to the small-genomed clades that may focus more on the abundant dissolved polysaccharide laminarin. Further to this, the *Alteromonadaceae* MAGs contain a greater diversity of PUL-like structures, as detailed in the Additional file 1: section *Alteromonadaceae*, which should allow consumption of either less soluble polysaccharides, or those that are only present at meaningful concentrations in particles.

While the other two groups, the *Halieaceae* and HTCC2089/*Pseudohongiellaceae*, also possess PUL-like structures, it is possible that these clades preferentially target other large biopolymers, of which we expect a major part is protein. The large numbers of TBDTs not co-located with CAZymes, demonstrated to be abundant in metaproteomes [35], is suggestive of this alternative focus for these clades. However, as mentioned above, genomic context rarely yields useful information. Additionally, numbers of peptidases per genome generally scale with genome size (data not shown). This is consistent with the idea that the proteins taken up by heterotrophs for anabolism and catabolism are a homogeneous mixture of peptide combinations that requires a single set of peptidases for cleavage. Lipid has also been suggested as an important source of energy for SAR86, based on proximity of putative lipid degradation genes to TonB-dependent transporters [39]. While we find several similar instances of TonB-dependent transporter proximity to glycine/betaine transporters and putative oxidoreductases, these, like the supposition of protein consumption, are less clear than the inferences regarding polysaccharide consumption.

Beyond the high molecular weight substrates, prospective capacity for consumption of dimethylsulfoniopropionate (DMSP), as indicated by DMSP demethylase (*dmdA* [44]) genes and the related 3-methylmercaptopropionyl-CoA ligase [45] and dehydrogenase functions [46], may be possible for species in the *Haliaeceae*, HTCC2089, SAR92, and SAR86, with the SAR86 genomes having two or often three of these genes. Several SAR92 also possess genes annotated as methylthioacryloyl-CoA hydratases typically also involved in DMSP consumption [46]. Proteorhodopsin genes are also present in MAGs from the HTCC2089, SAR92, UBA7434, and SAR86, but not in the *Halieaceae*, which are known to and here confirmed to be capable of anoxygenic photosynthesis [47, 48]. Both the DMSP degradation genes and proteorhodopsins are, however, subject to the same caveat that more in-depth confirmation would be required to truly confirm these observations.

### Unexpected functional diversity

**Fig. 4:**
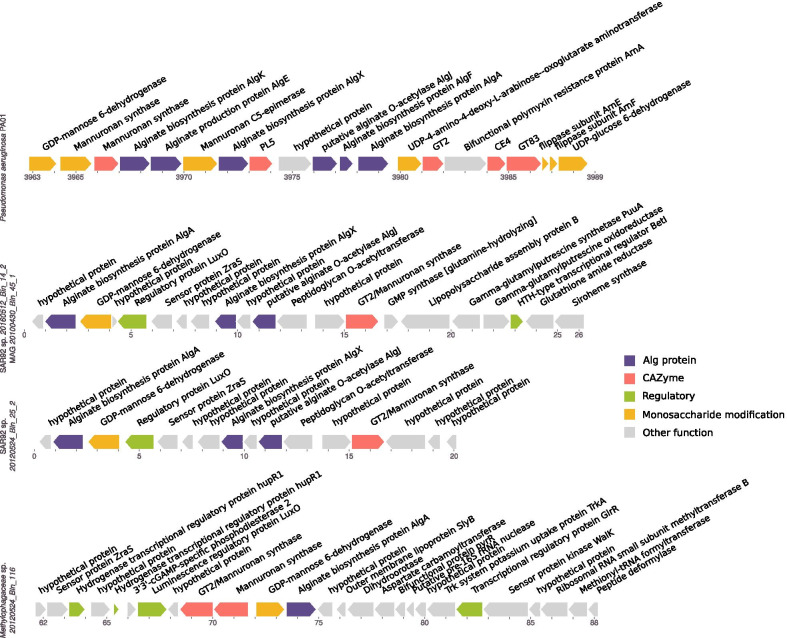
Gene arrangements of putative alginate synthesis loci in three North Sea Gammaproteobacteria species, and the characterised operon from Pseudomonas aeruginosa PA01. Tick marks indicate kilobase pairs from start of contig

In five SAR92 species, we identified homologues of the known alginate synthesis operons (Fig. 4), previously characterised in non-marine *Pseudomonas* and *Azotobacter* (for reviews see refs. [49–51]). These loci typically include the genes *algAEJX*, other cell wall and nucleotide sugar synthesis genes, and characteristically, genes encoding glycosyl transferases of the GT2 family identified as mannuronan synthases. Most interestingly, the highly abundant species *20160512_Bin_14_2* appears to possess this locus. The abundance of this species in 2010 (see Additional file 1: section SAR92) suggests that, if it were producing alginate at that time, it is possible that substantial amounts could have been produced, although we do not detect any proteins from this locus in the metaproteome data.

While the presence of predicted alginate degrading PULs identified here and in other studies [17] suggests alginate is an important polysaccharide degraded by bloom-associated bacteria, an obvious source of alginate is missing. To the best of our knowledge no microalgal producer has been identified. Meanwhile the brown macroalgae known to be alginate producers are present at Helgoland, but alginate release from them is not expected to coincide with phytoplankton blooms. Thus the prospect of bacterial production of alginate during blooms could be a solution to this problem.

The possible utility of alginate to the SAR92 is also not clear. It may be that it offers protection from grazing, viruses, other bacteria, or abiotic damage. Alternatively it may allow the bacteria to attach to surfaces or produce biofilms. The second of these hypotheses is, however, not consistent with the expectation based on size fractionated 16S rRNA gene amplicon analysis (Additional file 1: Fig. S3) that this clade has an almost exclusively free-living planktonic lifestyle, while nearly all genomes also show capacity for motility via flagella.

The other groups of species possessing predicted features of interest beyond polysaccharide degradation are the two sister families designated by GTDB as HTCC2089 and *Pseudohongiellaceae* (related clades are sometimes referred to as OM182—a name preserved as a genus in the *Pseudohongiellaceae* in the genome tree database (GTDB) [52] classification used in this study). The eight genera, all classified in GTDB with alphanumeric designations, have been little studied. These species have larger genomes, including in the HTCC2089 the largest genome in our gammaproteobacterial dataset, the 6.3 Mbp species *20160316_Bin_51*. Meanwhile their responsiveness to the blooms is also more unpredictable, even for the most abundant species, species *20100511_Bin_19_1* (see Additional file 1: section HTCC2089*/Pseudohongiellaceae*).

Gene annotations present in these species are similarly challenging. TonB-dependent transporters are typically present at lower density in the genomes than for the other clades in this functional group (Fig. 2), though still abundant in the metaproteomes. And as noted, these are primarily not associated with degradative CAZymes. But unusually, the genomes of the HTCC2089 clade do contain large numbers of annotated oxygenase genes. These are annotated with various functions whose specific substrates predicted by Prokka are likely to be unreliable. 92 genes across the 10 species are, for example, annotated by Prokka as linalool 8-monooxygenases. Pfam annotations almost exclusively label these under the more general function of Cytochrome P450. These may be involved in a variety of terpenoid oxidation reactions or oxidation of other aliphatic or aromatic compounds. The presence of the monooxygenases required for hydroxylation of hydrocarbons, coupled with alcohol dehydrogenases, aldehyde transforming aldehyde dehydrogenases, and ketone transforming Baeyer–Villiger monooxygenases, would all point to the possibility of complete degradation pathways for aliphatic compounds (for overview of pathways see [53, 54]). Other predicted substrates from Prokka, including dioxygenases active on biphenyl compounds, aromatic aldehydes, phenylacetone, anthranilate, and naphthalene, along with epoxide hydrolases, indicate a role in degrading aromatic compounds may also be possible.

There is also the possibility that these enzymes are responsible for demethylation of carbohydrates [55], or that they serve a role in biosynthesis of secondary metabolites (reviewed in refs. [56, 57]). However, given the numbers and diversity of monooxygenases predicted, and potential for complete oxidation, we suspect the most likely role for these bacteria is in the degradation of a wide variety of potentially algal secondary metabolites, or hydrocarbons from other sources, potentially including environmental pollutants.

Finally, nine of ten HTCC2089 species have co-localised homologues of at least two of the *glcDEF* genes, which are subunits of the glycolate dehydrogenase complex [58]. Glycolate has been shown to be produced in substantial quantities during phytoplankton blooms [18], and is thus an additional small molecule available to bacteria. This contrasts with the absence of DMSP degradation annotated in the HTCC2089 genomes, noteworthy given both glycolate and DMSP can be considered ‘algal small molecules’ with a common source. This raises the prospect of niche-partitioning between DMSP and glycolate degraders. Together these features suggest this clade has something of a crossover niche, combining growth on both small and large molecule components of algal organic matter.

## Small-molecule degraders

This grouping covers the 10 species of *Gammaproteobacteria* in our dataset in the clades *Nitrincolaceae*, *Saccharospirillaceae*, RS62, and *Thioglobaceae*; and the 21 species in the *Methylophilaceae*, CMS, SG8-40, and GCA-002705445 clades (Fig. 1). This collection includes clades that clearly increase in relative abundance in the metagenomes in response to the phytoplankton blooms, and those that either do not, or respond less strongly than the large molecule degraders. Again, we can divide the clades and identified functions into the expected and the unexpected. In terms of phylogenetic diversity, this collection covers a larger number of clades, some with proper names and some without.

### Expected diversity

Given the abundance of biopolymeric material produced by algae during spring blooms, we expect that the most abundant small molecules are likely to be the mono- or oligomeric amino acids, short chain peptides and small saccharides that polymers are composed of. These molecules are prospectively released as byproducts of the breakdown of polymeric material. The species that specialise on such substrates are few in number, but three groups, members of the *Nitrincolaceae*, *Saccharospirillaceae*, and RS62 clades, show clear responses in relative abundance during blooms, while the fourth, the *Thioglobaceae*, do not. These species have none or few TonB-dependent transporters, and instead possess in their genomes high densities of ABC transporters primarily for small peptides and amino acids (Fig. 2). The two standouts are the *Nitrincolaceae* species *20120531_Bin_63_1* and the *Saccharospirillaceae* species ‘*Reinekea forsetii’* [31], which both appear to respond later in the bloom period (*Reinekea* in two years, *Nitrincolaceae* in all four). Meanwhile, the RS62 stands out in having an apparent capacity for anoxygenic photosynthesis, like the *Luminiphilus* [48] (Additional file 1: Fig. S9). This is, to our knowledge, the first marine member of the former Betaproteobacteria to be identified to do this, however it has also been identified in the related freshwater genus *Limnohabitans* [59].

Only the *Thioglobaceae* and *Nitrincolaceae* MAGs among the small molecule degraders in our dataset have putative capacity for using DMSP, based on DMSP demethylase and DMSP lyase genes [60], the latter of which were not annotated in other clades. The more abundant *Nitrincolaceae* genome is also the only species with four annotated genes in tandem comprising two methanesulfonate monooxygenase hydroxylase subunits and two methanesulfonate monooxygenase ferredoxin subunits [61, 62]. Putative ABC-transporter subunits that might be involved in methanesulfonate uptake [63] were also present downstream of these genes in other MAGs from this species, although not in the species representative MAG. One route for DMSPdegradation produces gaseous dimethyl sulfide (DMS), which in turn is oxidised in the atmosphere to methanesulfonate [64, 65]. Methanesulfonate can then be returned to terrestrial and marine environments via precipitation and dry deposition [62, 66]. All four genes for the methanesulfonate monooxygenase are required for growth on methanesulfonate as a sole carbon source [61, 62], and its conversion as an intermediate in the assimilatory oxidation of DMS. The metaproteomic data indicates expression of these proteins in 2009 and less abundantly in 2016 (Additional file 1: Fig. S8). Much like the HTCC2089, both species of *Nitrincolaceae* also have co-localised homologues of the *glcDEF* genes, again suggesting some overlap in niche for the HTCC2089 between consumption of large and small molecules.

**Fig. 5:**
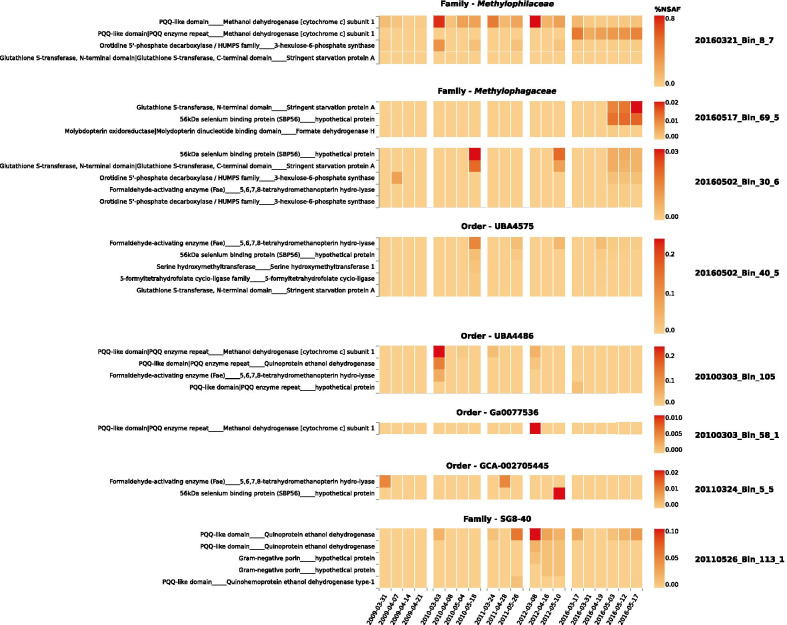
Expression in metaproteomes of methylotrophy related proteins among representatives from all of the clades predicted based on genome annotation to be capable of growth on C1 compounds, in particular methanol. Data is presented as %NSAF values, indicating the proportion of all protein in a sample. Data previously published in refs. [8, 9, 17, 35, 43]. Gene annotations to the left are first Pfam annotation, then Prokka annotation, separated by underscores "____"

The other small organic substrate known to be widely consumed by heterotrophic bacteria in these systems is methanol. It has been demonstrated that methanol is produced by algae [67], while one possible explanation for methanol production during spring blooms is the cleavage of methyl groups from polysaccharides as they are degraded (as mentioned above as a potential function of cytochrome P450 enzymes). Regardless of the source, it is clear that methanol is important to the North Sea heterotrophic bacterial community based on the five species of *Methylophilaceae* (OM43 group), and three *Methylophagaceae* (the ‘M’ in our CMS clade) in our data. These groups are known [68, 69], and confirmed by our genome annotations, to be capable of both assimilatory and dissimilatory use of methanol, via the RuMP pathway. The metaproteomic data also indicates that methylotrophic pathways are active during blooms, with expression of methanol dehydrogenases, glutathione S-transferases, SBP56 (methanethiol oxidase) [70], and the 3-hexulose-6-phosphate synthases involved in the anabolic RuMP pathway [71] (Fig. 5).

However, the *Methylophagaceae* are quite different from the *Methylophilaceae* in several ways. For example, their abundance was lower, they have larger genome sizes (1.9–2.2 Mbp versus 1.1–1.3 Mbp), and produced a more obvious bloom response than the *Methylophilaceae*. Gene annotations indicate the *Methylophagaceae* have more similarities with other general heterotrophs, such as genes for flagella, glycogen synthesis, type II secretion systems, and again unexpectedly, homologues for an alginate synthesis operon (Fig. 4). During spring blooms, however, metaproteomic data indicates that proteins involved in methylotrophy (Fig. 5) are the most prominent abundant proteins in these clades, with other abundant proteins including ribosomal proteins, DNA polymerases, ABC-transporters, and other general housekeeping proteins (Additional file 3: Table S2).

### Unexpected diversity of (facultative) methylotrophs

Perhaps the most unexpected outcome of our analyses is the wider diversity of methylotrophs present in these communities in addition to the *Methylophilaceae* and *Methylophagaceae* mentioned previously. The respective MAGs belong in most cases to order-level clades without proper names. These groups are the GTDB orders UBA4575, UBA4486, and Ga0077536 in the CMS clade, the basal order GCA-002705445, and the GTDB family SG8-40 in the *Burkholderiales* (formerly Betaproteobacteria). Specific details can be found in the individual subsections for these clades in the supplement, but from the metaproteomic data, it is clear that these clades engage in dissimilatory methylotrophy (Fig. 5), although it was not possible to conclusively identify either the RuMP pathway or another assimilatory pathway for C_1_ compounds. The SG8-40 and UBA4486 clades are also interesting in the abundant expression of methanol/ethanol family dehydrogenases [72], in contrast to the strict methanol dehydrogenases of the *Methylophilaceae*. Methanol is still the expected substrate consumed by these species, since ethanol is not known to be produced in any quantity during blooms.

There is also potential for consumption of other C_1_ compounds than methanol in these species. Methanethiol and methanesulfonate have been mentioned above, and proteins associated with their consumption are detected in the metaproteomic data, belonging to the *Methylophagaceae*, UBA4575, and GCA-002705445 (56 KDa selenium binding protein (SBP56) [70], Fig. 5), and the *Nitrincolaceae* (methanesulfonate monooxygenase, see Additional file 1: Fig. S8). Methylamine is another important C_1_ compound, however the genes for methylamine utilization [73, 74] are present in a more disparate set of genomes, namely the Ga0077536, several members of the HTCC2089 and *Pseudohongiellaceae*, and a *Luminiphilus* genome. Finally, the Ga0077536 species *20100303_Bin_58_1* possesses genes for the alpha, beta, and gamma subunits of a particulate methane monooxygenase complex [75–77], as do two other unusual but rare genomes: those of the *Acidovorax* and *Cycloclasticus*.

The Ga0077536 and *Cycloclasticus* genomes contain many remarkable features. The most prominent gene annotation in the *Cycloclasticus* species, and consistent with known activity of this genus on polyaromatic hydrocarbons [78–81], is the presence of eleven biphenyl dioxygenase complex genes, arranged in loci with genes for various other oxygenases, dehydrogenases, and dehydratases. Other genes with annotated functions include a toluene monooxygenase complex, and phenol hydroxylase proteins. The latter two of these are also present in Ga0077536 species *20100303_Bin_58_1*. The 4.9 Mbp Ga0077536 MAG *20100303_Bin_58_1,* by contrast contains the largest number of genes annotated as oxygenases of any of our MAGs at 286, compared to the 221 present in the next most oxygenase rich genome (HTCC2089 species *20160517_Bin_40_1*). The second Ga0077536 species (*20110321_Bin_75_1*) is, remarkably, only 2 Mbp in size and similarly complete (97% versus 98% for the larger species), but has 94 oxygenase genes annotated. Looking at just monooxygenase genes, the Ga0077536 genomes are even more impressive, with the ratio per Mbp for the two Ga0077536 species of 36 and 40 genes, far above the 22 for the next best HTCC2089 species. As with the HTCC2089 above, the most common Prokka annotations of these oxygenase genes are aldehydes and ‘limonene’. The presence of genes encoding alcohol dehydrogenases, alkanal monooxygenases, Baeyer–Villiger monooxygenases, epoxide hydrolases, and dioxygenases again indicates that more complete pathways for both linear and cyclic aliphatic hydrocarbons, and also aromatics, are present in these species, as they are for the HTCC2089.

Finally, unmentioned thus far, remain a collection of “others”, which are either not especially abundant during the blooms, and most likely do not participate substantially in the heterotrophic recycling of algal organic matter, or do not have features that make them stand out as much as those we have dwelt on here. Consequently, these clades are detailed only in the Additional file 1: subsection ‘Others’.

## Conclusions

As has been previously documented, the *Gammaproteobacteria* represent a large and heterogeneous part of the bacterioplankton community in coastal surface waters, both in terms of cell numbers and overall diversity [7–9]. It is clear that the bulk of the diversity in the samples we analysed—i.e. covering a small part of the pre-bloom period, and then the main part of the spring blooms themselves in the four years 2010–2012 & 2016—was made up of species that reach higher relative abundances over time in response to the growth of algae. We found fewer species of *Gammaproteobacteria* than *Bacteroidetes*, which may indicate higher levels of specialisation on individual substrates in that clade, such as has been proposed, for example, for *Polaribacter* species [82]. However, we have clearly identified a wider range of potential lifestyles, including growth on small organic molecules such as methanol and hydrocarbons, putative autotrophic and photoheterotrophic growth, and specialisation on amino acids, oligopeptides, sugar monomers, and oligosaccharides, in the *Gammaproteobacteria*. These prospective metabolisms offer up hypotheses including (1) that methanol/C1 compound production (and consumption) during phytoplankton blooms plays a more prominent role than previously thought. (2) That either hydrocarbon pollution or natural production of small hydrocarbons offers a substantial source of reduced carbon for specialised *Gammaproteobacteria*. (3) And finally we have the hypothesis that bacterial populations during spring blooms produce substantial amounts of alginate.

In conjunction with the *Bacteroidetes,* we see the main ecological role of the *Gammaproteobacteria* during phytoplankton blooms as primary remineralisers of larger polymeric organic matter such as polysaccharides and proteins. Where the boundaries lie in terms of the different substrates consumed is hard to say given the limited information we can glean from current ‘omics approaches. The obvious limitation here is the large number of TonB-dependent transporters we can annotate, but for which there is not an obvious candidate for the transported molecule. We do know, however, that these transporters should be quite specific for certain molecular species, hence the large numbers of genes detected. We presume, however, that the *Gammaproteobacteria* are less specialists for polysaccharides than are the *Bacteroidetes*. Furthermore, we also see several species that we predict are specialists for the many smaller molecules produced during spring blooms, that are not targeted by the *Bacteroidetes*. These species are fewer in number, most likely reflecting the lower complexity of the low molecular weight fractions. They nonetheless are significant, given, for example, the reports for example of high abundances of the *Nitrincolaceae* (and in particular the genus ASP10-02a) from locations in both Northern and Southern Hemispheres [8, 33, 34, 83].

Perhaps as is clear, when assessing numbers of genomes on the scale we have attempted here, it is only possible to scratch the surface. When each species or genus might justify its own publication, the task of condensing groups down to approximate categories, and even then only focusing on aspects of organic matter turnover, is absolutely necessary if one is to try to interpret a larger part of an ecosystem. However, we intend with this work to show that it is possible to learn something important and useful from such a dataset. Given the large numbers of metagenome assembled genomes (MAGs) that are now being extracted from vast sequencing projects that have been undertaken for so many environments, it is vital not just to generate genomes, but to attempt to understand their place in a biological context, i.e. to predict their niche in the bacterioplankton community.

Inevitably we are hampered by the limitations of annotation approaches and databases. Whenever there are hypothetical proteins in our gene annotations (frequently upwards of 40% of genes are annotated as hypothetical), we are completely blind. And although manual annotation is of course the gold standard, with so many genomes being generated, it becomes an enormous task that would have rendered our analyses nigh-impossible. However, in narrowing down niche-spaces of interest, the annotations worthy of the necessary additional in-depth domain-specific annotation and biochemical confirmation become clearer. And we consider it apparent from the above that we could indeed find some signal in the noise, and infer from our MAGs some general features for a large number of species that are present and abundant around the time of spring phytoplankton blooms in the North Sea. With these data we and others have a basis on which to begin that more detailed work on specific clades or functions of interest. We may still be looking through a foggy window, but at least now we’re looking during the daytime.

## Methods

### MAG data and choice of species representative MAGs

In this study we specifically analysed the previously published MAGs from Helgoland spring blooms during the years 2010, 2011, 2012, and 2016 that are in ENA project PRJEB28156 [17]. All of these data derive from prefiltered samples corresponding to the fraction of bacterioplankton in the size range of 0.2–3 µm. In order to delimit ‘species’ among these MAGs and make the analysis tractable by reducing redundancy, we used MASH v1.1.1 [84] to cluster MAGs into approximate species clusters with MASH distances shorter than 0.05 (approximately equivalent to 95% average nucleotide identity). From these species clusters we then selected the most complete MAG, or in certain cases a longer MAG (> 100 kbp additional length) with almost as high completeness and no additional contamination, to be the species representative for further analyses. This approach to redundancy removal inevitably results in the loss of some real diversity that will exist within MAGs species clusters, however this is a necessary and pragmatic cost in order to make useful analysis of the gammaproteobacterial part of the overall community feasible.

### Metagenomic read mapping to estimate abundance

Read mapping for the estimation of MAG abundance has been described before [17, 85]. Reads were mapped to individual species representative MAGs using BBMap v35.14 in ‘fast’ mode, with minid = 99 and idfilter = 97. Read counts were then converted to reads per kilobase per million (RPKM = ((reads mapped ÷ length of MAG in kbp) ÷ total number of reads sequenced) × 1 000 000), to account for the variation in MAG length and number of reads in each metagenomic dataset. In general, the RPKM values correspond to approximately double the percentage relative abundance values identified from direct cell counts, based on cell counts reported in ref. [8].

### Oligotyping to estimate abundance abundance from 16S rRNA gene amplicons

16S rRNA gene amplicon data used here has been reported previously [86–88]. Briefly, amplicons were generated for the V4 region of the 16S rRNA gene from samples collected after prefiltration through 10 µm pore size filters, via sequential filtration on 3 µm pore size polycarbonate filters, then on 0.2 µm pore size filters. Sequences were generated at the DOE Joint Genome Institute (JGI, Walnut Creek, CA, USA), where raw sequence data are also in the GOLD database with project IDs Gp0056779 (0.2–3 µm size fraction), and Gp0072732 and Gp0072733 (3–10 µm size fraction). Oligotyping was done using minimum entropy decomposition [89], with minimum substantive abundance (-M) of 100, and decomposition of one nucleotide position at a time (-d 1). Oligotypes were classified with the Silva v132 database implemented via the SilvaNGS web service, and mitochondria and chloroplast sequences were removed prior to abundance calculation.

### Genome annotation

In order to rapidly produce annotations for species representative MAGs, we annotated using Prokka v1.2 [90], and with Pfam v33.1 [91, 92], using the pfam_scan.pl tool and default settings. Additional annotation of CAZymes was done using dbCAN v6 [93] and DIAMOND BLAST v0.8.27 [94] against the CAZy database v07312018 [95]. Annotations were then viewed manually to find metabolism relevant to carbon cycling. Annotations of interest were manually confirmed using protein BLAST [96] via the BLAST web interface, and presumed to be annotated consistently by Prokka across species at least within the class *Gammaproteobacteria*.

### Phylogenetic reconstruction

Classification and phylogenetic reconstruction of representative MAGs was estimated using GTDB-Tk v0.3.1 and GTDB v89 [52]. GTDB-Tk incorporates pplacer v1.1 [97] for classification, HMMER v3.2.1 [98] for identifying single copy genes in MAGs, and FastTree v2. [99] for inferring phylogenetic trees.

Phylogeny for 16S rRNA genes identified in any of the PRJEB28156 MAGs was inferred using RAxML v8.2.9 [100], using the GTRGAMMA substitution model, and 1000 rapid-bootstrap subsamplings. Reference sequences were taken from Silva v138 [101], with sequences assigned taxonomy using SINA v1.6.1 [102].

### Metaproteome data

Metaproteome data used was previously published in refs. [8, 9, 17, 35, 43], and was dereplicated between the datasets where necessary when BLAST [96] identity between amino acid sequences exceeded 99%.

## Supplementary Information

**Additional file 1**. **Supplementary results.** Extended detail including abundances and further analysis of individual clades.

**Additional file 2.Table S1.** Completeness and contamination, and GTDB taxonomy for the 120 representative *Gammaproteobacteria* MAGs.

**Additional file 3**. **Table S2.** Metaproteome data from 20 samples from the years 2009-12 and 2016.

**Additional file 4**. **Table S3.** Gene annotations for the 120 representative *Gammaproteobacteria* MAGs.

## Data Availability

Metagenome data and metagenome assembled genome data used in this study are available in the ENA project PRJEB28156. All metaproteome data has been previously published, and is summarised here in Additional file 3: Table S2. Gene annotations used are presented in Additional file 4: Table S3.
