# Supplementary material for: North Sea spring bloom-associated Gammaproteobacteria fill diverse heterotrophic niches
Source: Environ Microbiome. 2021 Aug 17;16:15. doi: 10.1186/s40793-021-00385-y (PMC8371827; doi:10.1186/s40793-021-00385-y)
Supplement: Supplementary file 1 — Additional file 1. Supplementary results. Extended detail including abundances and further analysis of individual clades. [file 40793_2021_385_MOESM1_ESM.pdf]

**North Sea spring bloom-associated *Gammaproteobacteria* fill diverse heterotrophic niches**

## Supplementary text

## Results and Discussion

### 1. 16S rRNA gene based phylogenetic reconstruction

Of the 120 species, only 43 had at least one MAG with a 16S gene longer than 1000 base pairs. A number of these were found to be erroneous, either through binning errors or assembly error (presumed chimeric), and were removed from the subsequent tree (Supplementary Fig. S1). The phylogenetic reconstruction shows a relatively broad distribution of taxa from across the class *Gammaproteobacteria*. Well known groups such as the SAR92 clade and the *Alteromonadaceae* are well represented, as are the betaproteobacterial OM43 (*Methylophilaceae*) and RS62 clades, while lesser known but interesting groups such as Ga0077536 and *Methylophagaceae* (both CMS clade) are also detected. We also see that some of the groups in unnamed clades according to the GTDB-tk classification appear to fall within named groups in the 16S tree. The clearest examples are the GTDB family SG8-40 (16S represented in MAGs of species *20110526_Bin_113_1*), which according to its 16S rRNA gene sequence is in the *Nitrosomonadaceae* (genus IS-44); the species *20160502_Bin_40_5* in the order (and lower ranks) UBA4575, which appears closely related to *Candidatus* Tenderia; the species *20100413_Bin_38_1* in the order UBA10353 (lower ranks UBA7415), which appears closely related to the genus *Arenicella*, in the *Arenicellaceae*; the species *20110519_Bin_52_1* in the family (and lower ranks) UBA7434, which is closely related to the *Spongiibacteraceae* (genus BD1-7); and the only non-*Luminiphilus* species in the *Halieaceae*, species *20120412_Bin_16_1*, which appears to be most closely related to the genus *Halioglobus*.

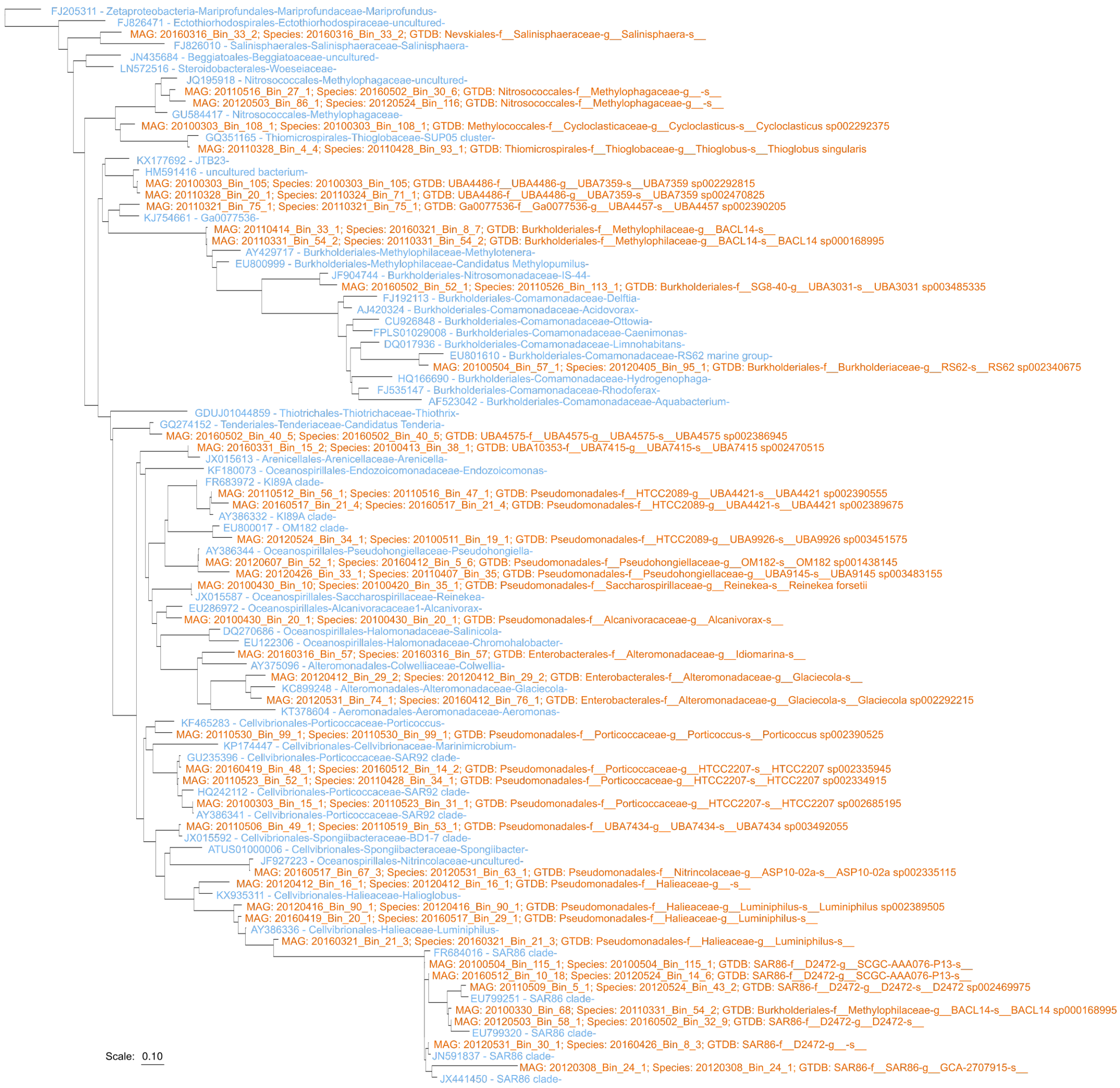


**Supplementary Figure 1.** 16S rRNA gene based phylogenetic reconstruction of gammaproteobacterial 16S sequences from MAGs. Orange sequences are from MAGs, with both the source MAG and species representative given, as well as GTDB taxonomy of the MAG. Blue sequences are from Silva v138, given with SINA v1.6.1 classification of the respective sequence.

## 2. Metagenomic read recruitment based clade abundance

The most abundant groups of *Gammaproteobacteria* during spring blooms at Helgoland were not always the most species rich (Fig. 1, main text). For detail of individual clade abundance,

see figures presented with each clade below. We observed high abundance of the SAR92 and SAR86 groups, (RPKM peaks up to 40 and 20 respectively, Fig. 1b, main text), whereas abundance of each of the *Alteromonadaceae*, *Pseudohongiellaceae*, and HTCC2089 families was less than 10 RPKM at peak abundance (Fig 1b, main text). *Halieaceae* abundance was similarly low with the exception of 2016 where RPKM peaked at almost 12, while the reverse was true for the *Methylophilaceae*, which had a peak near 20 in all years except 2016. The *Thioglobaceae* in every year dropped in relative abundance from a peak recording on the first sampling date (RPKM of 6 at its highest in 2011). This of course is not to say that the overall population size of this and other 'non-responding clades' was in decline, or even necessarily flat. Given the large increase in total bacterial cell numbers over the course of a spring bloom, these clades may increase in absolute number (i.e. respond to the bloom), but simply not as rapidly as other populations.

Other groups also appear to respond to blooms, reaching high RPKM values for single species that are approximately equivalent to 1% or more of the total community (RPKM above ~2). The most prominent are the more abundant of the two species in the family *Nitrincolaceae*, which in 2010 and 2011 reached RPKM of approximately 8; one species in the GTDB species, genus, and family UBA7434 which reached RPKM of 5-6 in 2010-11; and one species each of the genera RS62 and *Reinekea*, which both reached RPKM of 2 and above.

**Clade by clade detail**

Each clade described in detail here begins with a plot of the abundances of the MAGs belonging to that clade based on recruitment of metagenomic reads. Further description of important annotated functions and protein abundances follow.

*SAR92*

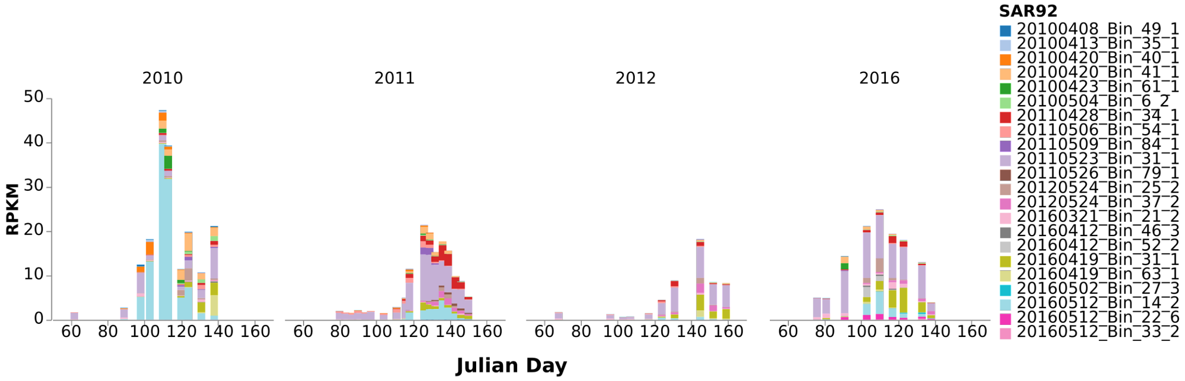


The SAR92 clade (GTDB genus HTCC2207) has been previously documented as being abundant and responsive during North Sea spring blooms [1–3]. With 22 species represented among our MAGs, it is the single most species rich gammaproteobacterial genus in the dataset, and is also the most abundant with a peak RPKM of 47 (20 April 2010). The peak was closer to 20 in subsequent years, implying a relative abundance of approximately 10% of the bacterioplankton community compared to ~23% in 2010. Two species, *20160512_Bin_14_2* in 2010, and *20110523_Bin_31_1* in the other years, were much more abundant than the other species and we consider the latter the archetype for the bloom-associated members of this genus. Genome sizes for the MAGs >90% complete (14 species) range from 1.6 Mbp to 2.9 Mbp (Supplementary Table S1; Fig. 1b, main text), and are 2.5 and 2.4 respectively for the two species just mentioned. This range is broad, but indicates these genomes are generally small, much like the putatively free-living bloom associated *Bacteroidetes*.

The SAR92 MAGs also appear similar to the *Bacteroidetes* in terms of inferred carbon acquisition strategy, suggesting the two groups may be in direct competition. Primary among them is the presence of many putatively large organic molecule specific TonB-dependent

transporters that should allow the ‘selfish’ sequestering of higher molecular weight substrates such as polysaccharides and proteins in the periplasm. The number of annotations of TonB-dependent transporter, vitamin B12 transporter BtuB, colicin and pesticin receptors, ferrichrome-iron receptor, ferripyoverdine and ferrienterobactin receptors in the SAR92 species range from 7.6-16.7 per megabase pair (13.6 in the archetypal species; Fig. 2, main text).

For the majority of these, a specific substrate cannot be predicted. However the SAR92 genomes do contain PUL-like CAZyme clusters (not necessarily co-located with transporters) where a polysaccharide degradation function can be putatively assigned. The main algal derived polysaccharide available to the bacterioplankton during phytoplankton blooms is the diatom storage polysaccharide laminarin, a β-1,3- and β-1,6- linked glucose polymer. Six of the SAR92 genomes have CAZyme clusters containing either a combination of GH16 and GH3 family genes (present in the archetype), or containing GH17 family genes (present in the other five) (Supplementary Fig. 2, below). Genes in these families in these combinations have been shown to be active in the degradation of laminarin in *Bacteroidetes* [4].

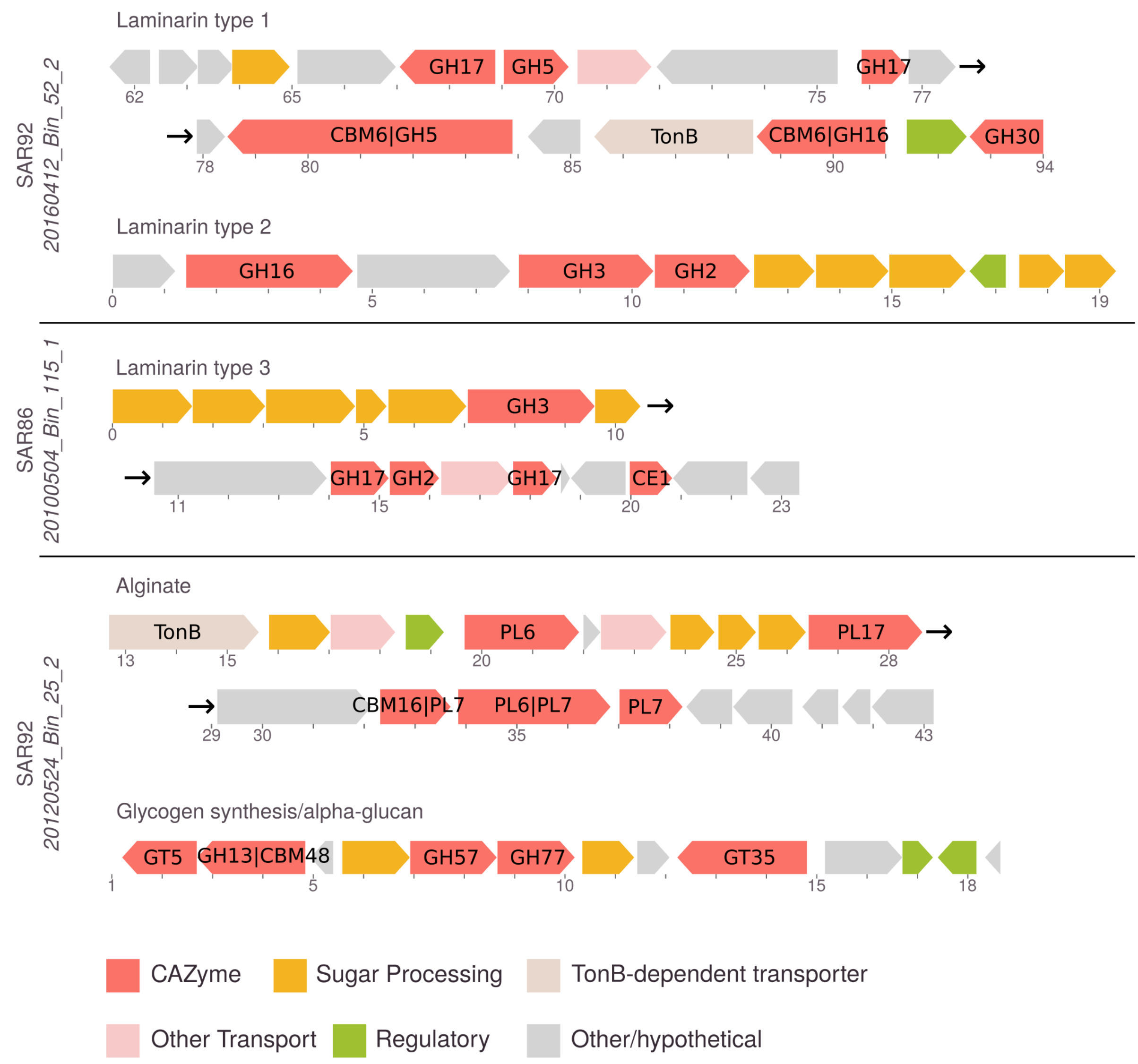


**Supplementary Figure 2.** Examples of common types of PUL gene arrangement in the SAR92 and SAR86 clades. Arrows (⟶) indicate continuation of the locus to the line below. Tick marks indicate position on contig in kilobase pairs for scale.

The next most frequent types of putative polysaccharide degrading gene clusters are those targeting α-glucans (found in four SAR92 genomes). These polysaccharides include starch, pullulan, and glycogen. The gene arrangements, featuring GH13, GH57, and GH77 family CAZymes, are distinct from those identified in *Bacteroidetes* that largely only contain GH13 family genes, and in greater numbers (Supplementary Fig. 2). It is likely that these gene

clusters in the SAR92 are involved in both synthesis and degradation of glycogen, and it is not easy to say if there is really consumption of externally sourced starch or pullulan produced by algae.

Finally, in three species we can predict alginate degradative function, on the basis of clustered PL6, PL7, and PL17 genes (Supplementary Fig. 2). It is surprising that beyond degradation of glucose-based storage polysaccharides, alginate should be the only other one predicted to be consumed by the SAR92. It has been seen to be an important and widespread target for the bloom-associated *Bacteroidetes* [5], but in that study the algal cell wall-associated mannose and xylose rich polymers were considered the more significant. It appears therefore that SAR92 do not use homologous genetic componentry to directly compete with *Bacteroidetes* for access to the algal cell wall.

The source of alginate putatively consumed by these bacteria is also somewhat mysterious, since to the best of our knowledge no microalgal producer has been identified. The brown macroalgae that are known to be producers are present at Helgoland, but it is not clear that release of their alginate into solution should coincide with phytoplankton blooms. However, metaproteomic data suggests that active uptake (inferred from detection of the PUL-associated putatively alginate-specific TonB-dependent transporters) is occurring during spring blooms[6]. In four representative SAR92 genomes we identified homologues of known alginate synthesis operons characterised in non-marine *Pseudomonas* and *Azotobacter* (Fig. 4, main text). These loci typically include the genes *algAEJX*, other cell wall and nucleotide sugar synthesis genes, and characteristically, genes encoding glycosyl transferases in the GT2 family identified as mannuronan synthases. Among all SAR92 MAGs, we found five belonging to species *20160512_Bin_14_2* also had the same gene cluster. The abundance of

this species in 2010 means it could have been a significant source of alginate if it did indeed produce it. The purpose of this alginate is not one that we can readily speculate upon. Possible hypotheses could be that it offers protection from grazing, viruses, other bacteria, or abiotic damage. Alternatively it may allow the bacteria to attach to surfaces or produce biofilms, however neither of these would be consistent with the expectation that this clade has a primarily free-living planktonic lifestyle (Supplementary Figure 3, below).

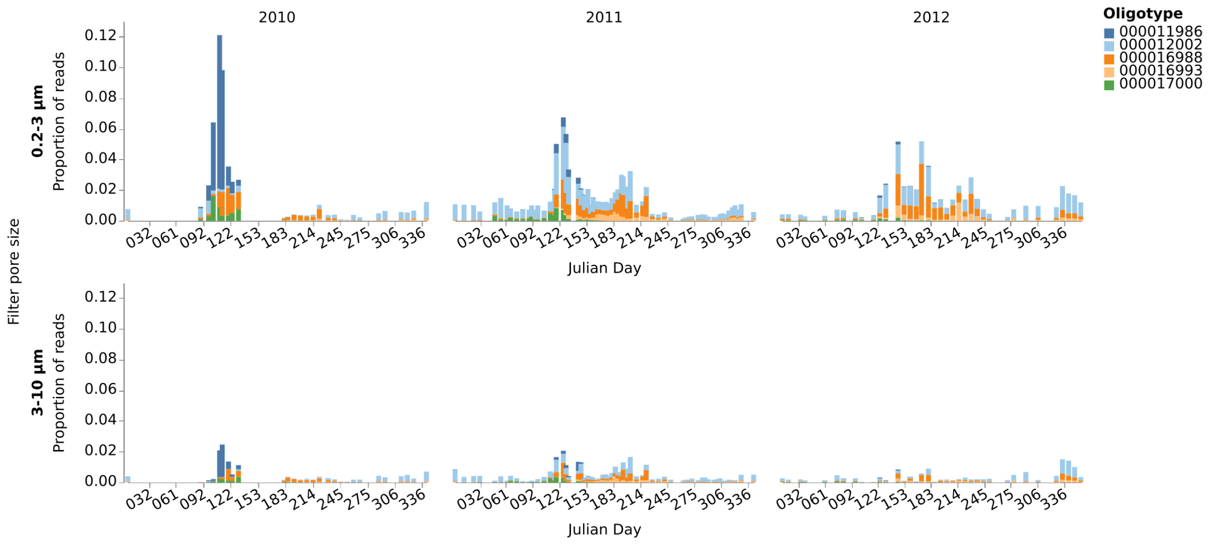


**Supplementary Figure 3.** Abundance of SAR92 classified 16S rRNA amplicon oligotypes over the full years 2010-12 for two size fractions approximately corresponding to 'free-living' (upper row), and 'particle-associated' (lower row). Data are filtered to include only oligotypes reaching above a proportion of 0.01 of all reads on at least one date.

Besides putative polysaccharide degradation, there is little of note among the SAR92. There is a good chance that the abundant TonB-dependent transporters in their genomes are used to transport larger organic substrates of unknown composition, though quite likely proteins feature among them. Peptidase frequencies and families are rarely specific enough to identify substrates however, since the linkage types in peptides are much less diverse than for sugars.

Additionally there is no reason to doubt that these species can make use of amino acids and small peptides, sugar monomers and small oligosaccharides. All but three representative MAGs have annotated proteorhodopsin genes, indicating potential for use of light energy is widespread in this clade, as it is in many others. Fifteen of the species also have genes encoding flagella, indicating widespread swimming motility in this clade, which is a clear contrast to the aflagellate *Bacteroidetes*.

*SAR86*

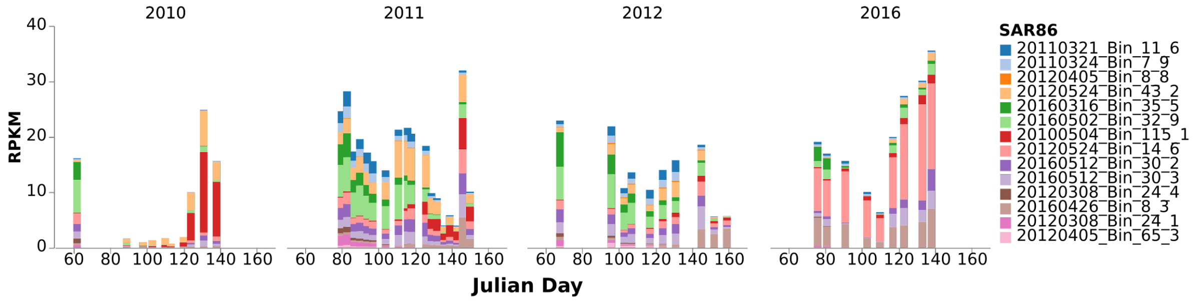


MAG studies of SAR86 have already established the primary features of this ubiquitous clade [7]. First and foremost they have smaller genomes than the SAR92, with consequently streamlined predicted metabolism which nonetheless tilts toward the consumption of larger organic molecules such as putatively carbohydrate. The analysis of our 14 phytoplankton bloom associated SAR86, 12 of which are in the GTDB family D2472, is largely consistent with previous reports. There is evidence for nitronate consumption based on the presence of nitronate monooxygenase genes, and nitroreductase genes, DMSP consumption based on presence of dimethylsulfonioproprionate demethylase genes (*dmdA*), and the presence of proteorhodopsin genes [7].

The genomes are rich in TonB-dependent transporters (range 9.3-17.0 per Mbp), similar to the SAR92 (Fig. 2, main text). In contrast, we find little in the way of PUL-like structures, except in four of the most clearly bloom-responsive species (*20100504_Bin_115_1*,

*20120524_Bin_14_6*, *20160426_Bin_8_3, 20160512_Bin_30_3*), which each have characteristic putative laminarin degrading CAZyme clusters including GH17 and GH3 family genes (Supplementary Fig. 2, above). In the other bloom-responsive species (*20160512_Bin_30_2*, *20120524_Bin_43_2*), however, we see no such structures. Again there is the possibility that other larger organic molecules are consumed, particularly proteins. In ref. [7] it was suggested lipid may also be an important source of energy for SAR86, based on proximity of putative lipid degradation genes to TonB-dependent transporters. While we find several similar instances of proximity of glycine/betaine transporters, and putative oxidoreductases, these remain speculative assignments of function, much like that of protein transport.

One final noteworthy feature is the slightly higher number of peptidases among the SAR86 genomes, which range from 51 to 69 peptidases per Mbp. It could be argued that smaller genomes inevitably have a higher ratio of peptidases simply to maintain normal cellular function. However, the contrast with other clades with small genomes may suggest otherwise. The three SAR86 MAGs with completeness estimates above 90% have total lengths in the region of 1.5 Mbp, while there is one *Thioglobus* species, two *Methylophilaceae* species, and two species in the order GCA-002705445 that have genome sizes below 1.2 Mbp. The three SAR86 genomes have 55, 56, and 61 peptidases per Mbp, while the other five are in the range 41-51. This is at least consistent with the hypothesis that there is a link between TBDT number, peptidase number, and protein consumption as a strategy.

*Halieaceae*

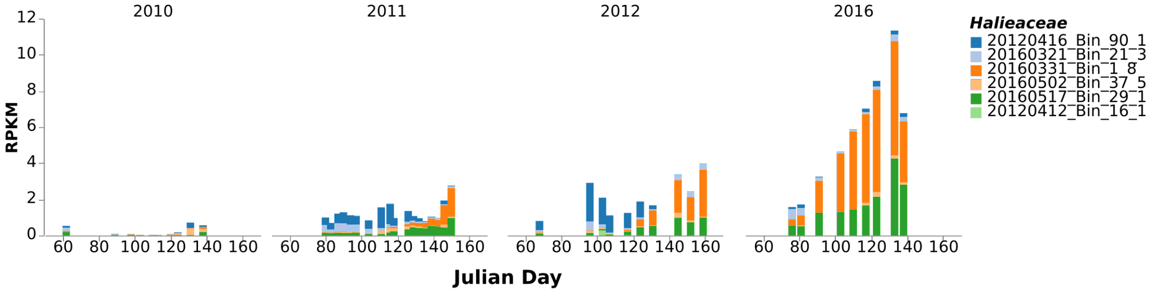


Five of the six *Halieaceae* species in our data belong to the genus *Luminiphilus*, also sometimes known as NOR5/OM60. The sixth, present at very low abundance and the only one from this family with completeness below 90%, appears based on the 16S rRNA gene phylogeny to be related to the genus *Halioglobus* (Supplementary Fig. 1). Genome sizes range from 2.5-3.1 Mbp for *Luminiphilus*, while the less complete *Halioglobus* relative is 4 Mbp.

Three *Luminiphilus* species appear to peak late during blooms, with relative abundance of two of them (*20160331_Bin_1_8*, *20160517_Bin_29_1*) on an upward trend through the end of our sampling periods in all years, and the third (*20160502_Bin_37_5*) just beginning to decline. In contrast, the species *20120416_Bin_90_1* has an earlier peak, while the less abundant species *20160321_Bin_21_3* appears not to respond in 2010 and 2011, but does so late in 2012 and 2016. The *Halioglobus* relative appears to respond mid-bloom, but to no more than 0.1% of the total bacterioplankton.

Similar to the SAR92 and SAR86, the *Halieaceae* species are rich in TBDTs (range 9.9-20.8 per Mbp - Fig. 2, main text). The *Halioglobus* species in fact records the highest TBDTs per Mbp and highest total number - 84 - of any species in our study. PUL-like CAZyme clusters are even less frequent in these species, however. Species *20160331_Bin_1_8* has a single locus with two GH17 family genes and a GH5 family gene annotated, while species *20120416_Bin_90_1* has two sets of GH16 and GH3 family genes, one with a predicted

TBDT. Both of these are expected to be laminarin targeting gene clusters similar to those in Supplementary Fig. 2. Again this leaves us with little in the way of clear knowledge of substrates, and the transporters are co-located with many different annotations. As previously mentioned for SAR86, protein or fatty acids are eminently plausible candidates. We thus conclude that these species, much like the SAR92 and SAR86, are specialists for larger organic molecules. These diverse and difficult to interpret gene clusters surrounding TonB dependent transporters are similar to some of those shown in Fig. 3 in the main text.

An additional putative carbon source for these species is DMSP, based on *dmdA* genes which are found in four of the five *Luminiphilus*. The other noteworthy feature of the *Luminiphilus*, as indicated in the name and described previously [8], is their capacity for anoxygenic photosynthesis (Supplementary Fig. 4, below). We do not see this feature in the *Halioglobus* species.

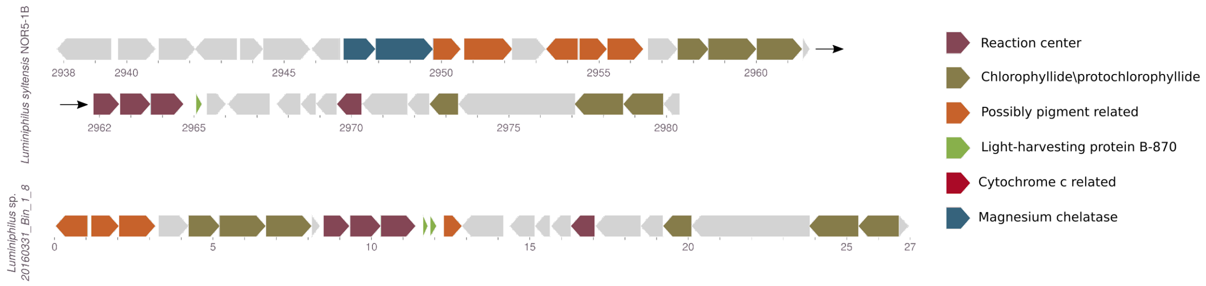


**Supplementary Figure 4.** Gene arrangements of putative aerobic anoxygenic photosynthesis enabling gene clusters from reference *Luminiphilus syltensis* NOR5-1B and *Luminiphilus* sp. *20160331_Bin_1_8*. Tick marks indicate kilobase pairs from start of contig. Arrows (⟶) indicate line breaks, the gene arrangements themselves are contiguous.

At least some of the reaction center genes were also found to be expressed by three different species during periods of high abundance in 2016 (Supplementary Fig. 5, below), indicating these species actively engage in anoxygenic photosynthesis during spring blooms.

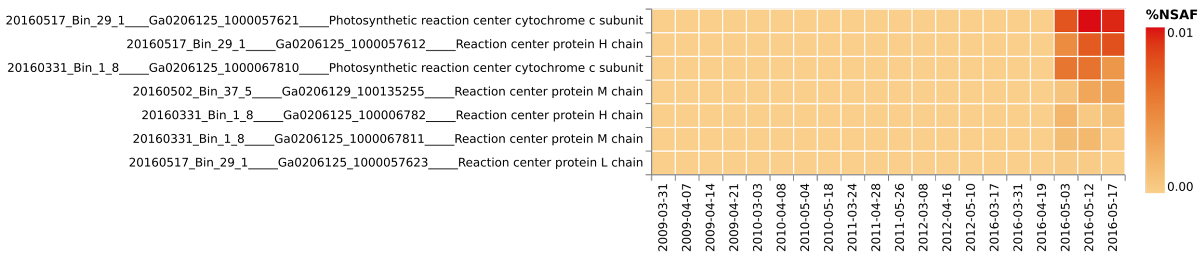


**Supplementary Figure 5.** Metaproteome abundance of photosynthetic reaction center proteins belonging to species of the genus *Luminiphilus* during spring blooms in the years 2009-12 and 2016. Protein abundance is given as normalised spectral abundance factor (%NSAF), which indicates protein abundance as a proportion of all detected protein in a sample. Labels are species representative MAG, protein accession, and Prokka annotation (Pfam annotation corroborates all seven Prokka annotations).

*HTCC2089/Pseudohongiellaceae*

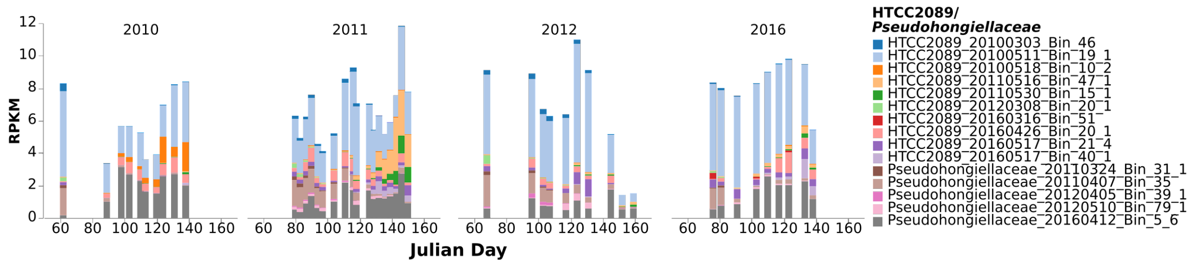


The two sister families designated by GTDB as HTCC2089 and *Pseudohongiellaceae* (related clades sometimes referred to as OM182 - a name now preserved as a genus in the *Pseudohongiellaceae*) are somewhat difficult to interpret. There are multiple genera, all classified in GTDB simply with alphanumeric designations, and there appears to have been little previous characterisation of these groups. In general genome sizes are slightly larger

than the groups previously mentioned. Of the ten genomes estimated to be more than 90% complete, only two of the *Pseudohongiellaceae* genomes are below 3 Mbp, while the HTCC2089 include the largest genome in our gammaproteobacterial dataset, the 6.3 Mbp species *20160316_Bin_51*.

Several species show clear bloom-response peaks, while others follow the non-responsive pre-bloom community pattern. Among the *Pseudohongiellaceae*, only the OM182 species *20160412_Bin_5_6* responds to blooms, while in the HTCC2089 only two species consistently do not. However several appear to respond only in some years, and the most abundant species, *20100511_Bin_19_1*, shows rather erratic abundance patterns that are not easily interpreted.

Following that theme, the gene annotations present in these species are similarly challenging. TonB dependent transporters are once again a clear feature, but at lower frequency than the previous clades (Fig. 2, main text). For the *Pseudohongiellaceae*, the range is 6.8-11.3, and for the HTCC2089, 5.5-12.0. Again prospectively polysaccharide targeting PUL-like gene clusters are rare, with just one each of predicted laminarin and α-glucan degrading regions identifiable in the 5 representative genomes in this family.

This may mean their strategy is more diversified away from relying solely on larger organic matter. However, these species seem not to have more di-/tri- and oligopeptide ABC transporters than the SAR92 per Mbp of genome (range 1.1-4.5 against 1.0-3.0 for SAR92). A similar pattern is observed when we look at transporters for amino acids and amino acid-like molecules such as putrescine, spermidine, cadaverine, or taurine. Here the range for SAR92 (0.8-5.1) again overlaps with that for the *Pseudohongiellaceae*/HTCC2089 (0.5-3.0).

Where the groups diverge, however, is in perhaps the most mysterious feature of the HTCC2089 clade genomes: their large numbers of annotated oxygenases. These are described in detail in the main text, and comprise potential for complete degradation of both aliphatic and aromatic hydrocarbons.

Finally, nine of ten HTCC2089 species have co-localised homologues of at least two of the *glcDEF* genes, which are subunits of the glycolate dehydrogenase complex. Glycolate has been shown to be produced in substantial quantities during phytoplankton blooms [9], and is thus an additional small molecule available to bacteria. It is interesting to contrast this with the absence of DMSP degradation annotated in the HTCC2089 genomes however, since both glycolate and DMSP can be considered 'algal small molecules' where we might expect both to be consumed since they have a common source.

*UBA7434*

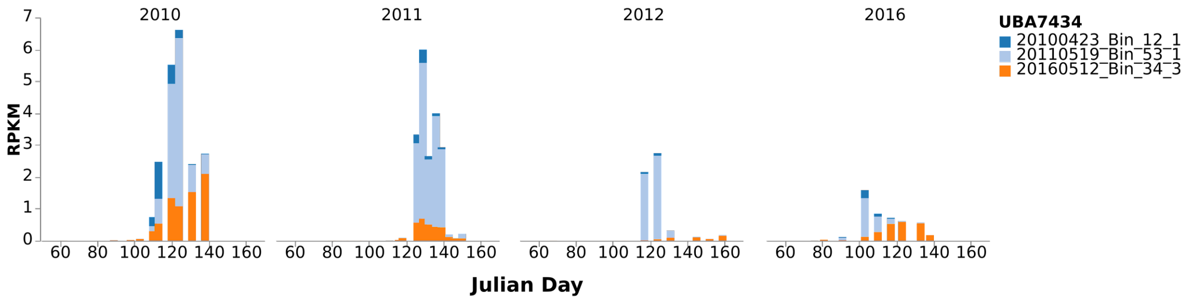


The UBA7434 clade, a sister taxon to the *Halieaceae* and *Oceanicoccus*, is much like the HTCC2089 in having been little studied beyond basic phylogeny. All three species in our dataset appear to respond to phytoplankton blooms, in quite narrow mid-bloom peaks.

This clade has TonB-dependent transporters (8.3-10.7 per Mbp), but not unusually many (Fig. 2, main text). One noteworthy characteristic is the presence in one of the three species (*20110519_Bin_53_1*) of a putative sulfated xylan-degrading locus (GH10, GH10/GH16, GH3, GH5, sulfatases, Supplementary Fig. 6). This and another species (*20160512_Bin_34_3*) also have a locus targeting an unidentified substrate requiring sulfatases, GH136 family genes, and GH3 family genes (example from species *20110519_Bin_53_1* shown in Supplementary Fig. 6, below). The GH136 family has to date only a single characterised CAZyme from *Bifidobacterium longum*, with lacto-N-biosidase function [10]. PULs in the *Bacteroidetes* with similar composition are not uncommon, and a similar locus is present in SAR92 species *20120524_Bin_25_2*.

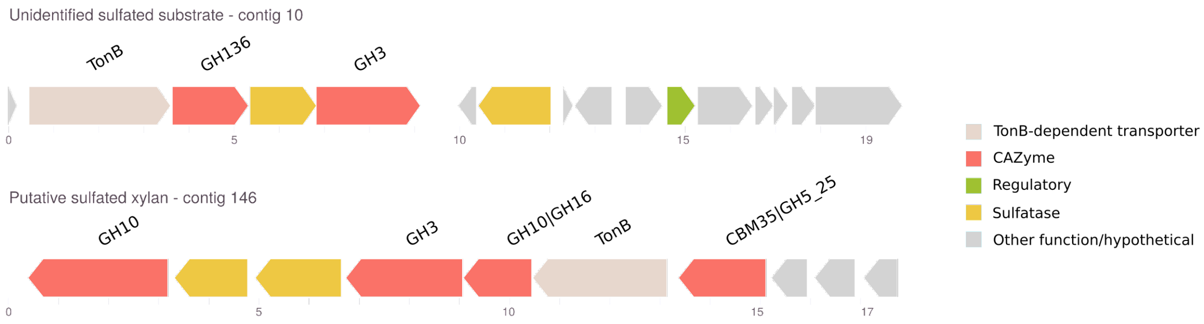


**Supplementary Figure 6.** Putative sulfated polysaccharide utilisation loci in UBA7434 species *20110519_Bin_53_1*. Tick marks indicate kilobase pairs from start of contig. Arrows (⟶) indicate line breaks, the gene arrangements themselves are contiguous.

The lack of CAZyme clusters for the more abundant glucose polymers is peculiar among our *Gammaproteobacteria* that appear to be more polysaccharide oriented. It is also the case that the putatively sulfated xylan degrading locus is on a relatively short contig, and could be misattributed to this species as an artefact of the binning process. Sulfated xylans have

nevertheless been predicted to be important to the *Bacteroidetes* during phytoplankton blooms, and it is interesting that this is the only instance identifiable among the *Gammaproteobacteria* MAGs where we predicted the targeting of this substrate.

*Alteromonadaceae*

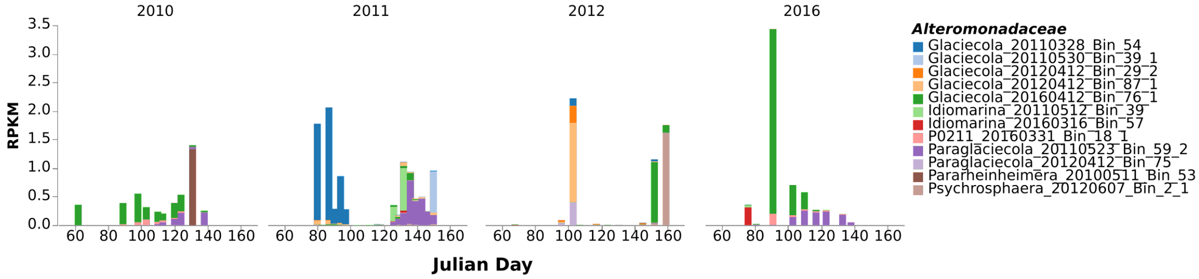


The *Alteromonadaceae* are outliers in our dataset in that they are species rich without being very abundant. Of twelve species, nine are predicted to be more than 90% complete on both metrics employed. Genome sizes are in general larger than for the more abundant species - with the exception of the two *Idiomarina* species and one *Glaciecola*; all those more than 90% complete are larger than 3 Mbp in length, and five of those nine span more than 4 Mbp. With genomes of this size, we expect that these species are less likely to be a primary part of the 'free-living' community, and instead more likely associate with particles and surfaces, or possibly sediments, and thus would have wider potential metabolic range adapted to localisation within a spatially narrow but nutrient rich environment. Consistent with this, the one *Glaciecola* species with a genome size below 3 Mbp, species *20160412_Bin_76_1*, was the most abundant of the *Alteromonadaceae* (peak RPKM = 3) in the metagenomic data, corresponding to the smaller 'free-living' 0.2-3 µm fraction, in 2016 than any other species in this family managed in any year. Looking at the larger size fraction meanwhile, there appears in general higher relative abundances of *Alteromonadaceae* than in the smaller fraction (Supplementary Fig. 7). For example, the species *20110328_Bin_54*, with a 4.3 Mbp genome, appears to have been present at the same time in 2011 as the oligotype 14089 in

Supplementary Fig. 7. This oligotype, which we infer comes from the same species, made up as much as 30% of the sequences from the larger size fraction during this period, compared to perhaps 1-2% of the smaller size fraction.

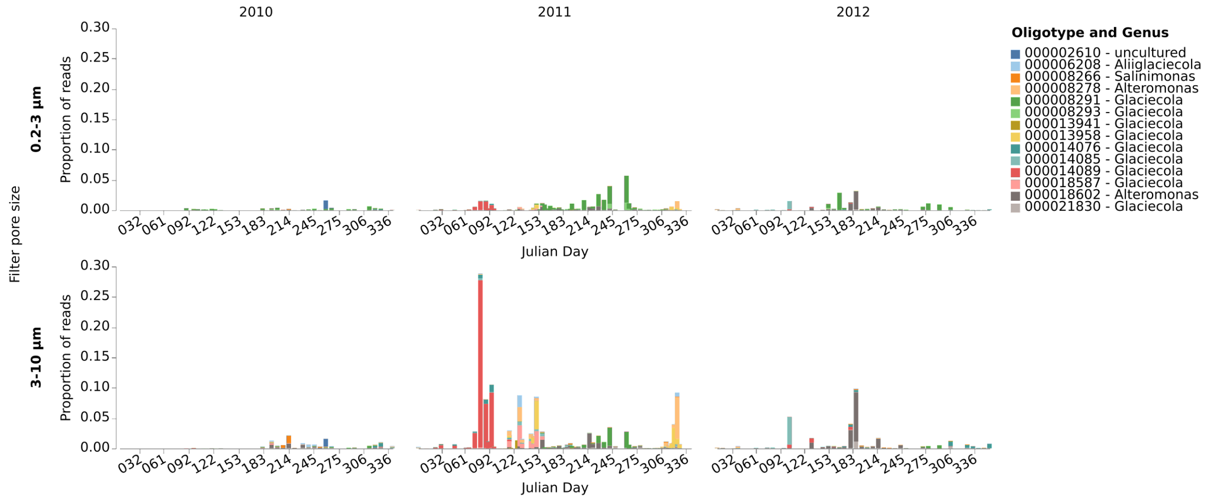


**Supplementary Figure 7.** Abundance of *Alteromonadaceae* classified 16S rRNA amplicon oligotypes over the full years 2010-12 for two size fractions approximately corresponding to 'free-living' (upper row), and 'particle-associated' (lower row). Data are filtered to include only oligotypes reaching above a proportion of 0.01 of all reads on at least one date.

Also informing our expectation for an in general more particle associated lifestyle, the gene annotations for the *Alteromonadaceae* indicate a greater focus on polysaccharide degradation, and for the most part greater diversity of function, than for the other gammaproteobacterial clades. The more clearly bloom responsive *Paraglaciecola* species *20110523_Bin_59_2* has PUL-like loci predicted to be for laminarin and α-glucan degradation, which should be the most consistently predictive for bloom-response in the bacterioplankton. The other *Paraglaciecola* species (*20120412_Bin_75*), in contrast looks more likely to attack particles, since it has PUL-like loci predicted to degrade mannose-rich polysaccharide (multiple GH92 family genes), chitin (multiple CBM5/AA10 domains, 2 × GH18), an unidentifiable substrate

(possibly ulvan related?) requiring GH105, GH88/PL5 and GH2 CAZymes and a TonB-dependent transporter, a rhamnose-rich polymer (multiple GH78 and sulfatases), and several loci for α-glucan.

*Glaciecola* species (five in total), meanwhile, are well equipped with putative laminarin and α-glucan targeting genomic loci. Additionally, they have loci predicted to target alginate, inulin/levan (GH32/CBM38, and a rare gammaproteobacterial *susCD*/*ragAB* transporter pair), a potentially rhamnose-rich polymer (GH145, GH28, GH105), the same speculatively ulvan-like substrate as mentioned above in *Paraglaciecola*, and mannose rich polysaccharide (GH130/GH76, GH5). Finally among the *Alteromonadaceae* species that have identifiable PUL-like structures, is the *Pararheinheimera* species. This species has a putative pectin degrading locus (PL10, CE12, CE8, GH105, GH28), a putatively chitin targeting locus (GH18, CBM5/AA10), two putative xylan-degrading loci (GH10/GH9, GH115, GH43; GH8, GH11/CE6, GH3, GH43), and a putative mannan-targeting locus (multiple GH92, GH2, and GH85). The other species in the *Alteromonadaceae* (the two *Idiomarina* species, a *Psychrosphaera* species, and a species in the genus P0211) do not appear to have PUL-like structures in their genomes, except for a possible arabinan-targeting locus (GH43, GH127/GH146) in *Idiomarina* species *20160316_Bin_57*, present on a short contig. These latter four species each have smaller genomes than the *Glaciecola* and *Paraglaciecola* species, which in part relates to completeness in the case of the two non-*Idiomarina*. But it has been demonstrated that *Idiomarina* species show signs of genomic streamlining [11], which would appear from the genome sizes to be in effect here as well. However, with neither species producing regular bloom responses nor presence at high abundance during blooms, whatever streamlining may have occurred seemingly does not permit rapid growth on algal biomass.

*Nitrincolaceae*

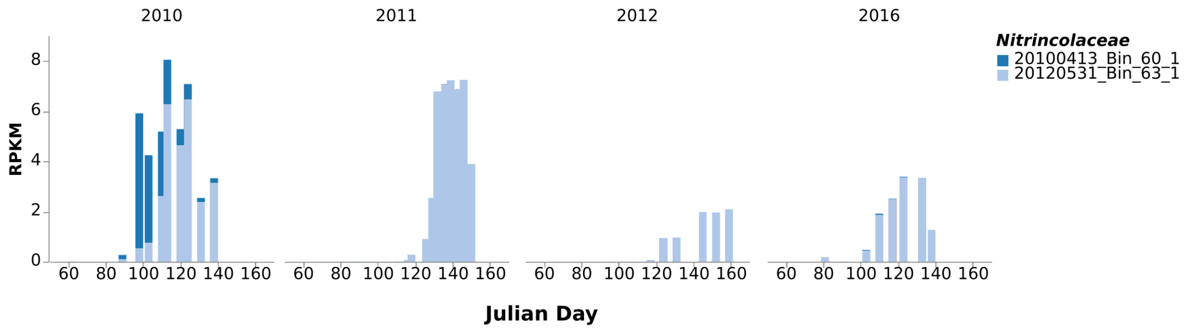


The two species in our dataset in the family *Nitrincolaceae* are both members of the genus level clade ASP10-02a. Previously this clade was recognised as somewhat distantly related to the genus *Balneatrix* [2]. Other studies have identified species in this genus as present during phytoplankton blooms in both northern and southern phytoplankton blooms at higher latitudes [12,13], and have speculated on possible interactions with phytoplankton based on these species' ability to produce vitamin B12 [12], which was annotated in our MAGs as well.

The two genomes are both near complete, at 2.2 and 2.6 Mbp for the less and the more abundant, respectively. The abundance of the larger genome is in the range of 1-4% of the bacterioplankton in all years, and always peaks in the latest phase of the bloom each year. The less abundant species was only detected in 2010, and in that year peaked somewhat earlier than its relative.

The two genomes are similar in gene content, with the more abundant species *20120531_Bin_63_1*, being simply more pronounced than the less abundant species *20100413_Bin_60_1*. The genomes of the two species in this clade feature a high frequency of transporters for small peptides and amino acids. Additionally, they have many transporters for sugar monomers, and among the highest for all species. For peptide transporters

annotated, species *20120531_Bin_63_1* has the highest proportion of any of our species, with 6.8 per Mbp. And similarly it possesses almost the highest density of amino acid and amino acid-like molecule transporters, with 19.7 per Mbp. For sugar monomer transporters, the number is 3.6 per Mbp. For ABC transporters as a whole the figure is 39.0 per Mbp, higher than any other gammaproteobacterial species in our data.

The more abundant species has six dimethylsulfoniopropionate demethylase (*dmdA*) genes, as compared to two or three in other species. It is the only species with four annotated genes in tandem comprising two methanesulfonate monooxygenase hydroxylase subunits and two methanesulfonate monooxygenase ferredoxin subunits. DMSP when degraded produces dimethyl sulfide (DMS) which in turn is broken down to methanesulfonate. All four genes for the methansulfonate monooxygenase are required for growth on methanesulfonate as a sole carbon source[14], and its conversion as an intermediate in the assimilatory oxidation of DMS.

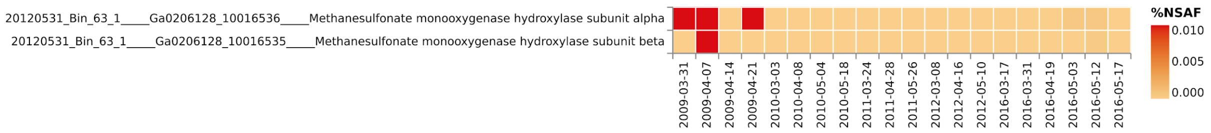


**Supplementary Figure 8.** Metaproteome abundance of the alpha and beta subunits of the methanesulfonate monooxygenase hydroxylase in *Nitrincolaceae* species *20120531_Bin_63_1* during spring blooms in the years 2009-12 and 2016. Protein abundance is given as normalised spectral abundance factor (%NSAF), which indicates protein abundance as a proportion of all detected protein in a sample. Labels are species representative MAG, protein accession, and Prokka annotation. Methanesulfonate monooxygenase ferredoxin subunits neighbouring the hydroxylase in the genome were not detected in the metaproteome.

Finally, both species of *Nitrincolaceae* have co-localised homologues of the *glcDEF* genes, much like the HTCC2089. Once again this highlights the distinct focus of these species on the small molecules rather than polymers. The complete absence of TonB-dependent transporters in these genomes is further testament to that inference (Fig. 2, main text). It is entirely plausible that such a strategy could result in close relationships with the algae themselves, since free amino acids are likely to be utilised by phytoplankton-associated bacteria once released, as could DMSP be, thus being ‘fed’ in return for vitamins would make sense. This is a lifestyle often predicted for members of the *Alphaproteobacteria*[15,16], with which the *Nitrincolaceae* may therefore compete. The abundance over time of the *Nitrincolaceae* group doesn’t speak to this hypothesis however. One might expect a higher abundance earlier in the bloom if the bacteria were associating with living algae, and the late peak could in contrast mean they only have sufficient resources to flourish once almost all the algae are dead. It may be that the substrates preferred by these species are not abundant enough until most algal cells have died. Alternatively, sugar monomers, small peptides, and amino acids deriving from cleavage of proteins and polysaccharides may accumulate as they are degraded by the polymer specialists.

*RS62*

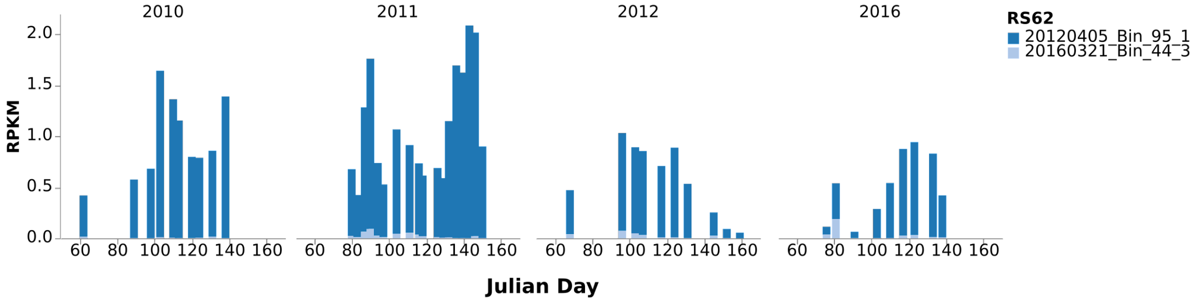

The RS62 clade is the one of three noteworthy betaproteobacterial clades, along with the *Methylophilaceae* and SG8-40. In terms of abundance, the species *20120405_Bin_95_1* is the more significant of the two, reaching approximately 1% of the overall community in 2011 (RPKM ~ 2). This species is also estimated to be very nearly complete at 2.5 Mbp, whereas the species *20160321_Bin_44_3* genome is less than half as long, and estimated at only ~50% complete.

Gene annotations indicate a similar focus on amino acids and amino acid-like molecules as seen in the *Nitrincolaceae* and *Thioglobaceae*, with 24.4 and 26.3 transporters per Mbp in the more complete and less complete genome respectively. ABC transporters as a whole have densities of 28.8 and 38.0 respectively. There is also a large skew in the annotated functions of the amino acid transporters, with 57% the Prokka annotations comprising the two high-affinity branched-chain amino acid transporters livF and livH. In the *Nitrincolaceae* these annotations made up 22% of the amino acid transporters, and in the *Thioglobacaeae* 4%. There are no indications for the consumption in these species of other small molecules that have been mentioned previously, such as DMSP, glycolate, or aromatic compounds. The genomes also lack identifiable TonB-dependent transporters.

What we do see, however, in the more complete genome, is the full complement of genes predicted to code for the anoxygenic photosynthesis pathway mentioned above for the *Halieaceae* (Supplementary Fig. 9, below). The sequences in the RS62 species have high similarity to the equivalent genes in the freshwater betaproteobacterial genus *Limnohabitans* (80-94% amino acid sequence identity) [17]. To our knowledge this is the first such identification of this function in a marine betaproteobacterial species.

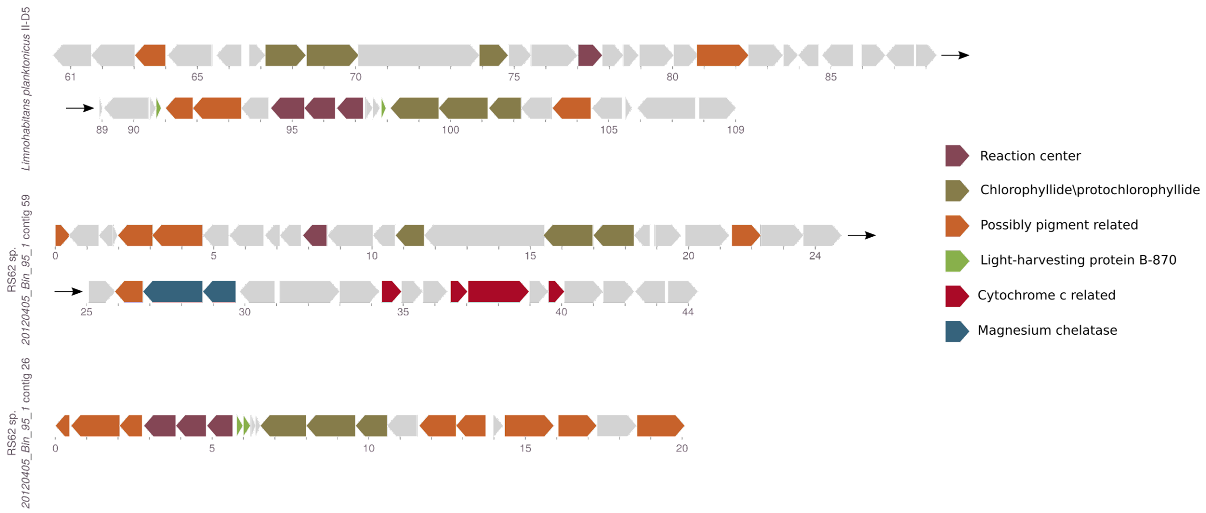


**Supplementary Figure 9.** Gene arrangements of putative aerobic anoxygenic photosynthesis enabling gene clusters from reference *Burkholderiales* species *Limnohabitans planktonicus* II-D5 and RS62 sp. *20120405_Bin_95_1*. Tick marks indicate kilobase pairs from start of contig. Arrows (⟶) indicate line breaks, the gene arrangements themselves are contiguous.

As was the case for the *Luminiphilus* species, these proteins are also detectably expressed during the blooms (Supplementary Fig. 10).

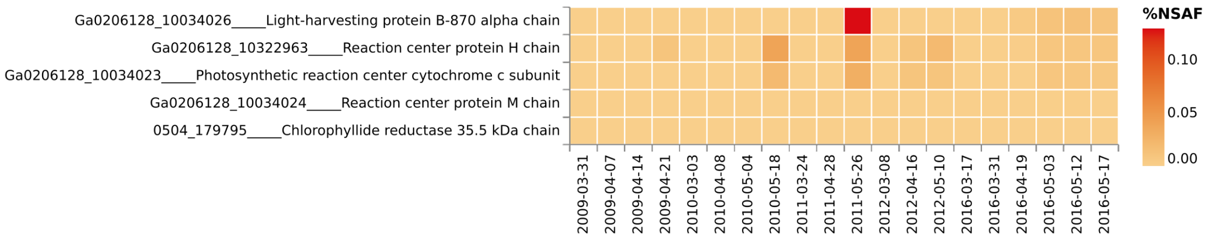


**Supplementary Figure 10.** Metaproteome abundance of photosynthetic reaction center proteins belonging to RS62 species *20120405_Bin_95_1* during spring blooms in the years 2009-12 and 2016. Protein abundance is given as normalised spectral abundance factor (%NSAF), which indicates protein abundance as a proportion of all detected protein in a sample. Labels are protein accession and Prokka annotation (Pfam annotation corroborates all Prokka annotations).

*'Reinekea forsetii'*

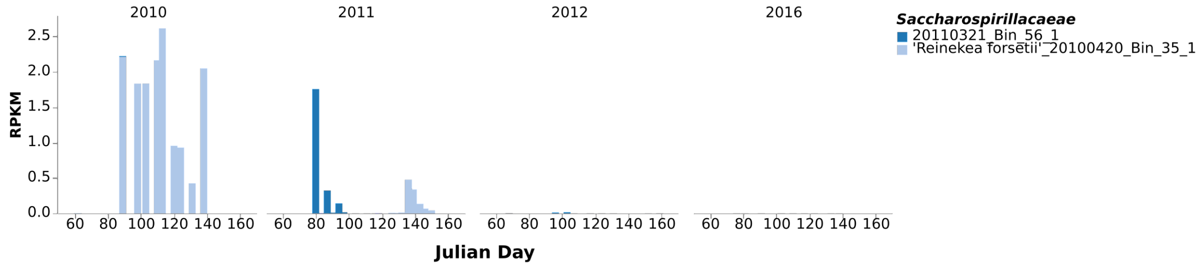


The single species of *Reinekea*, in the family *Saccharospirillaceae*, '*R. forsetii'*, has been studied in detail based on a cultured population previously [18], although has not been validly published and is therefore in quotation marks. The MAG species is highly similar in nucleotide identity to that strain (>99%), however some analysis is worthwhile to place it in the context of the other clades already discussed. The species was abundant only in 2010, with a small peak in 2011, then not at all in 2012 and 2016. However, it was clearly bloom-responsive. It shares with the *Nitrincolaceae* an apparent preference for small peptides, having just one TBDT, while it has 5.8 predicted di-, tri-, and oligopeptide transporters per Mbp of genome. However, it has many fewer predicted amino acid and amino acid-like molecule transporters - 6.4 per Mbp - than the 20+ per Mbp found in the *Nitrincolaceae*. Again, in common with the *Nitrincolaceae*, it has many sugar monomer transporters - 5.1 per Mbp of genome. ABC transporters in general have a density of 32.0 per Mbp. It therefore seems the strategy of '*R. forsetii'* is somewhat similar to that of the *Nitrincolaceae* and the RS62, but lacking the amino acid transport capacity, as well as, for example, glycolate and DMSP consumption capabilities.

*Methylophilaceae*

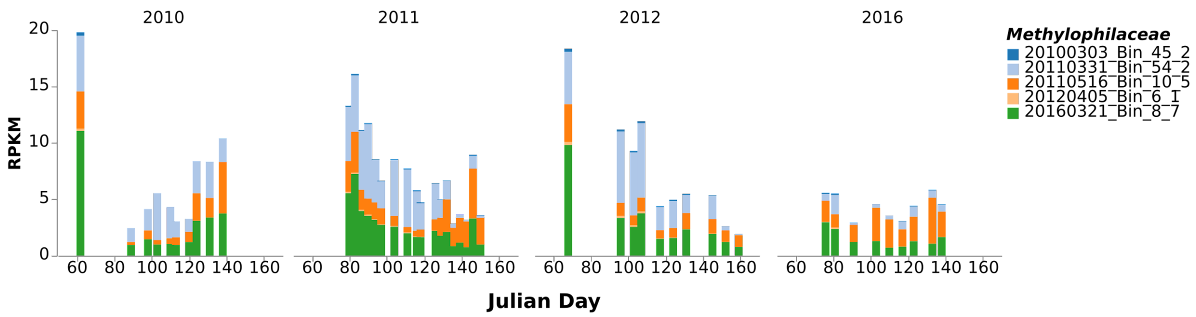


The five species in the *Methylophilaceae*, often also referred to as the OM43 clade, all have small genomes - the two most complete stand at 1.2 Mbp and 1.1 Mbp. These are the only two with genome completeness estimates above 90% (Supplementary Table S1). The five species are apparently a substantial component of the pre-bloom community - peak RPKM is usually on the first sampling date and reaches about 20 (~10% of the bacterioplankton) for the clade as a whole at least in the years 2010-12. However, interestingly, their relative abundance then follows a more 'U' shaped pattern over the course of the bloom. This recovery pattern contrasts with other pre-bloom or non-responsive clades, which fall to low relative abundances that stay low through the end of the sampling periods. This perhaps indicates the *Methylophilaceae* species are responding to the blooms, but at some time-delay relative to the faster growing species that consume polymeric material.

As should be clear from the name, and other literature on the OM43 group[19,20], these species specialise on single carbon compounds - predominantly methanol in marine surface waters. It has been demonstrated that methanol is produced by algae [21], and this is therefore consistent with the idea that the *Methylophilaceae* species are bloom-responsive. The central gene involved is the methanol dehydrogenase, present in all five of our species. This enzyme produces formaldehyde from methanol, which is then fed into the RuMP cycle for either assimilatory or dissimilatory oxidation. The 3-hexulose-6-phosphate synthase and isomerase genes, a 6-phosphogluconate dehydrogenase, and glucose-6-phosphate isomerase and

dehydrogenase genes associated with this pathway are present in all five species. Expression of both methanol dehydrogenases and key enzymes in the RuMP pathway was detected in the metaproteomes as well (Fig. 5, main text).

*CMS clade*

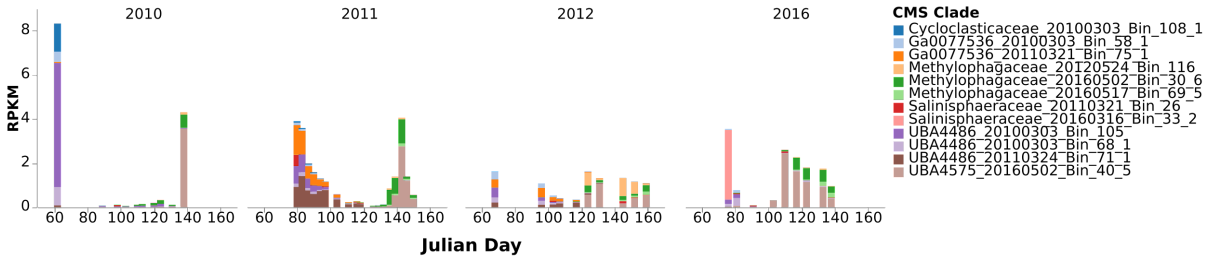


The broad monophyletic group we are terming the CMS clade includes substantial levels of diversity, much of which has not previously been studied in detail. The group as a whole comprises eight genera and twelve species in its six families and orders. Only one genome is estimated to be less than 90% complete (*Salinisphaeraceae* species *20110321_Bin_26*), and only one (Ga0077536 species *20100303_Bin_58_1*), at 4.9 Mbp, is larger than 3 Mbp in length.

For the most part, these species are pre-bloom non-responders (Fig. 6), with two exceptions being the trio of *Methylophagaceae* species, and the UBA4575 species. The two other named families, the *Cycloclasticaceae* and *Salinispharaceae*, are both only present at very low abundances, but for single early samples in 2010 and 2016, respectively. The more clearly pre-bloom community associated species are in the UBA4486 and Ga0077536 clades. These five species show abundance patterns similar to the *Methylophilaceae* in the early bloom phases, with steep smooth declines as a proportion of the overall bacterioplankton from initial highs approaching one percent of the bacterioplankton.

In spite of these differences in abundance patterns, there is much that unites these clades. Primary among them is an apparent ability among all but the *Salinisphaeraceae* and *Cycloclasticaceae* to oxidise formaldehyde via the tetrahydromethanopterin pathway. However, only the *Methylophagaceae* possess annotated genes for the RuMP cycle for assimilation of formaldehyde and single carbon compounds. Additionally, the UBA4486 species *20110324_Bin_71_1* and Ga0077536 species *20100303_Bin_58_1* are both predicted to have methanol dehydrogenase genes, further suggesting single carbon metabolism for these two groups.

Beyond methylotrophy, the CMS clade species have genes annotated for diverse small molecule degradation functions, similar to those mentioned above for HTCC2089. The most prominent in the *Cycloclasticus* species, and apposite given the name and known activity of the genus in simulated oil spill mesocosm experiments[22–25], is the presence of twelve biphenyl dioxygenase complex genes, arranged in loci with various other oxygenases, dehydrogenases, and dehydratases. Additional annotated functions for this genome include a toluene monooxygenase complex, phenol hydroxylases proteins, and the two subunits of a particulate methane monooxygenase/ammonia monooxygenase. Each of these is also present in Ga0077536 species *20100303_Bin_58_1.*

The 4.9 Mbp Ga0077536 MAG *20100303_Bin_58_1* contains the largest number of annotated oxygenases of any of our MAGs at 286, which is 29% more than the 221 present in the next most oxygenase rich genome (HTCC2089 species *20160517_Bin_40_1*). The second Ga0077536 species (*20110321_Bin_75_1*) is, remarkably, only 2 Mbp in size and similarly complete (97% versus 98% for the larger species), but has 94 oxygenases annotated. This is the third highest ratio of oxygenases per megabase pair - 46.5 - after the 58.8 of the other

Ga0077536 species and the 49.8 of HTCC2089 sp. *20160517_Bin_40_1*. The *Cycloclasticus* MAG has 33.6 oxygenases per megabase pair. Looking at just monooxygenases, the distinction is even starker, with the ratio per Mbp for the two Ga0077536 species of 35.6 and 39.7 more than double the 14.5 for the *Cycloclasticus* and 50% more than the 22.3 of the HTCC2089 species. Again the most common annotations of these oxygenases are aldehydes and 'limonene', i.e. probably terpenoids, for the Ga0077536, and biphenyl compounds for the *Cycloclasticus* species.

Both Ga0077536 species have a variety of other interesting gene annotations. One is the many annotated subunits of acetophenone carboxylases. The larger of the two MAGs has 12, while the smaller has 17. These are mostly in gamma-delta subunit pairs or as solitary delta or gamma genes, but in the smaller genome there is a complete 5 subunit locus annotated. The smaller genome also has four annotated acetone carboxylase subunits, three of them in an alpha-beta-gamma tandem[26]. Similar acetone carboxylase gene trios are present in the UBA4486 MAGs, the *Cycloclasticus* MAG, and the *Salinisphaeraceae* MAG *20110321_Bin_26*.

Both Ga0077536 genomes also contain the three methylamine utilisation protein genes *mauDEG*, and glycolate utilisation gene pairs similar to those seen in the *Nitrincolaceae*, and which are also present in the three UBA4486 species. All of these annotations suggest that these species are capable of consuming a considerable variety of aromatic compounds, for which there are not clear and defined sources.

*Thioglobaceae*

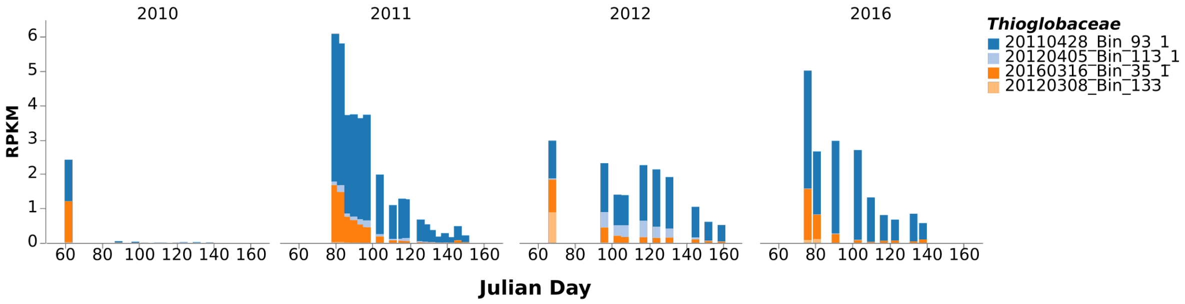


Of four species in our data that belong in the *Thioglobaceae* (part of the SUP05 cluster of marine thiotrophs), only one - *20120308_Bin_133* - has a representative MAG that is above 90% complete. This species is, however, neither the most abundant, nor was it detectable on more than a handful of sampling dates. The more representative species, with peak abundances reaching 1% of the bacterioplankton and more, are the *Thioglobus singularis* population represented by MAG *20110428_Bin_93_1*, and the *Thioglobus* sp. *20160316_Bin_35_1*. Both are seemingly non-responsive to blooms, with a clear decline in relative abundance in all four years. The abundance pattern of species *20120405_Bin_113_1* appears in 2010 and 2012, and to a lesser extent in 2016, to be more bloom-responsive with an initial increase in abundance, albeit to relatively low maxima.

The species in this family have similar carbon acquisition strategies to some of the other previously mentioned clades. Specifically, they have similarly higher per megabase pair rates of amino acid and small peptide transporters, though not as extreme as the *Nitrincolaceae* (ABC transporters, Fig. 2, main text), however the smaller and less complete genomes of the *Thioglobaceae* mean the absolute numbers are rather lower (amino acid transporters: 25 in the *Thioglobus singularis* MAG versus 52 in the *Nitrincolacaeae* MAG *20120531_Bin_63_1*, for ratios of 20.0 and 19.7 per Mbp respectively). There are also genes annotated in these species for the utilisation of methylamine, DMSP, and prospectively glycolate, in common with the *Nitrincolaceae* and certain members of the CMS clade. It is interesting that these

species are so clearly pre-bloom non-responders as compared to the *Nitrincolaceae*, given at least a superficially similar predicted focus on amino acids and small peptides. This may be a result of the small and streamlined genomes of the *Thioglobaceae* being more adapted to oligotrophy and survival rather than rapid growth.

The final aspect of the *Thioglobaceae*, unique among the *Gammaproteobacteria* in our dataset, is their capacity for chemolithoautotrophy. Annotated genes for sulfide oxidation and carbon fixation using RuBisCO are present in the two larger genomes (*Thioglobus singularis* and *20120308_Bin_133*). This ability is unlikely to be of great significance during the spring bloom sampling periods, where we expect the *Thioglobaceae* to be primarily heterotrophic and net remineralisers of carbon, but there is a clear source of hydrogen sulfide at Helgoland where anaerobic degradation of large piles of dead macroalgae on beaches occurs, and the resultant sulfide is washed out into the water.

*Others*

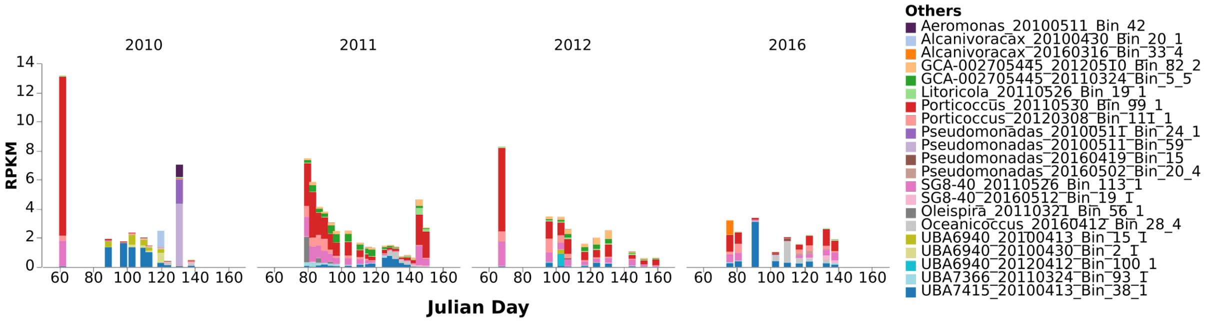


The close relatives of the SAR92 in the genus *Porticoccus* are both pre-bloom non-responders in 2010 and 2012, but follow the pattern of some of the *Methylophilaceae* in 2011 and 2016, initially dropping before later in the bloom producing an abundance peak (Fig. 6). Both genomes are near complete, the more abundant *20110530_Bin_99_1* being substantially smaller at 1.3 Mbp than the 2.1 Mbp *20120308_Bin_111_1*. Neither species has

many TBDTs, with three and five annotated respectively. Nor do they have many oxygenases nor large numbers of amino acid or peptide transporters. The only really noteworthy annotations are full operons of acetone and acetophenone carboxylase subunits, implying potential for growth on these two substrates.

Among the other clades not already mentioned, we see a distinct additional abundance pattern in addition to the bloom-responsive/non-responsive patterns covered earlier. Many species in this collection have single abundance peaks limited to individual sampling points. Many of the genomes are estimated to have high completeness, however, with thirteen of twenty above 90% according to at least one method, and a further four more than 80% complete. The four clades with single day abundances are *Alcanivorax*, *Acidovorax*, *Aeromonas*, and *Pseudomonas*. It is not obvious the reason for these clades' detection in our samples - they are generally marine species rather than soil or human associated, indicating they are unlikely to be contaminants. Whatever the reason for their detection, their abundance patterns suggest they have little to do with organic matter cycling in this environment.

Naturally of greater interest are those that are detected across multiple sampling dates. The non-responders include firstly the MAG of *Oleispira antarctica*, only detected in 2011, over four sampling dates early in the bloom. Then there are the two species in the betaproteobacterial family SG8-40, one of which - *20110526_Bin_113_1* - was much more abundant than the other. These follow the pattern mentioned above of steep decline, but which is followed in 2011 and 2016 by a late increase in relative abundance. Then finally there is the species in the order, family, genus, and species UBA7366, which declines in 2011, but is otherwise less abundant in other years but shows small increases in relative abundance over the sampling period. Apart from the SG8-40, mentioned in the main text as

novel methylotrophs, these species are not especially unusual or interesting in their carbon acquisition strategies.

Finally there are the species that appear to react to blooms, but less strongly than those mentioned earlier. The most abundant of these is the species *20100413_Bin_38_1*, in the family and genus UBA7415, a sister taxon to the betaproteobacteria according to our phylogenetic reconstruction, which reached RPKM of ~2 in 2010 and ~4 in 2016. This MAG contains a predicted laminarin-type PUL-like gene cluster and a GH32 rich putatively levan or inulin degrading gene cluster, and has many TonB-dependent transporters (8.2 per Mbp of genome - similar to SAR92 and others). The *Oceanicoccus* species *20160412_Bin_28_4* similarly has a laminarin-type gene cluster, along with an α-glucan type cluster, and 6.4 TBDTs per Mbp. This species reached a maximum RPKM of ~1.5 in 2016, with peaks of ~0.5 in 2010 and 2011.

The other clades that respond, *Litoricola* sp. *20110526_Bin_19_1*, the two species in the order GCA-002705445, and three species in the family UBA6940, all have low numbers of TBDTs. The *Litoricola* species looks more similar to the *Nitrincolaceae*, with higher numbers of amino acid and sugar monomer transporters (25.1 ABC transporters per Mbp). It also has genes for DMSP consumption, and unusually among our gammaproteobacteria, all five genes necessary for the BHAC (β-hydroxyaspartate cycle) pathway of glycolate degradation more commonly found in the *Alphaproteobacteria*[9], in addition to genes for the glycolate dehydrogenase complex.

The two species in the order GCA-002705445 both have very small genomes of 1.1 Mbp length. Both have complete sets of genes for the tetrahydromethanopterin pathway for

formaldehyde oxidation, indicating that they most likely operate in a similar mode to the other methylotrophic species already mentioned, however, we were not able to identify unambiguous assimilatory pathways for single carbon compounds via either the serine pathway or the RuMP pathway in these species.

Finally, the UBA6940 species are rather mysterious. Two species, with genome sizes of 3.2 and 2.3 Mbp (94% and 75% complete respectively) responded to the bloom in 2010, then a third (3.4 Mbp genome, 99% complete) did in 2012. These species have minimal numbers of amino acid transporters, sugar monomer transporters, oxygenases, no annotated methylotrophic pathways, and as mentioned, very few TBDTs. Furthermore none of their proteins were detected in the metaproteomes. It is thus largely unclear what might have fuelled their response to the spring blooms in the years they did.

## References


1. Needham, D. M. & Fuhrman, J. A. Pronounced daily succession of phytoplankton, archaea and bacteria following a spring bloom. *Nat. Microbiol.* **1**, 16005 (2016).
2. Teeling, H. *et al.* Recurring patterns in bacterioplankton dynamics during coastal spring algae blooms. *eLife* **5**, e11888 (2016).
3. Teeling, H. *et al.* Substrate-controlled succession of marine bacterioplankton populations induced by a phytoplankton bloom. *Science* **336**, 608–611 (2012).
4. Unfried, F. *et al.* Adaptive mechanisms that provide competitive advantages to marine *Bacteroidetes* during microalgal blooms. *ISME J.* **12**, 2894–2906 (2018).
5. Krüger, K. *et al.* In marine *Bacteroidetes* the bulk of glycan degradation during algae blooms is mediated by few clades using a restricted set of genes. *ISME J.* **13**, 2800–2816 (2019).
6. Francis, T. B. *et al.* Changing expression patterns of TonB-dependent transporters

suggest shifts in polysaccharide consumption over the course of a spring phytoplankton bloom. *ISME J.* 1–15 (2021) doi:10.1038/s41396-021-00928-8.

7. Dupont, C. L. *et al.* Genomic insights to SAR86, an abundant and uncultivated marine bacterial lineage. *ISME J.* **6**, 1186–1199 (2012).
8. Fuchs, B. M. *et al.* Characterization of a marine gammaproteobacterium capable of aerobic anoxygenic photosynthesis. *Proc. Natl. Acad. Sci.* **104**, 2891–2896 (2007).
9. Schada von Borzyskowski, L. *et al.* Marine *Proteobacteria* metabolize glycolate via the β-hydroxyaspartate cycle. *Nature* **575**, 500–504 (2019).
10. Sakurama, H. *et al.* Lacto-N-biosidase encoded by a novel gene of *Bifidobacterium longum* subspecies longum shows unique substrate specificity and requires a designated chaperone for its active expression. *J. Biol. Chem.* **288**, 25194–25206 (2013).
11. Qin, Q.-L. *et al.* Trophic specialization results in genomic reduction in free-living marine *Idiomarina* bacteria. *mBio* **10**, e02545-18 (2019).
12. Bertrand, E. M. *et al.* Phytoplankton–bacterial interactions mediate micronutrient colimitation at the coastal Antarctic sea ice edge. *Proc. Natl. Acad. Sci.* **112**, 9938–9943 (2015).
13. Delmont, T. O., Eren, A. M., Vineis, J. H. & Post, A. F. Genome reconstructions indicate the partitioning of ecological functions inside a phytoplankton bloom in the Amundsen Sea, Antarctica. *Front. Microbiol.* **6**, 1090 (2015).
14. Higgins, T. P., Davey, M., Trickett, J., Kelly, D. P. & Murrell, J. C. Metabolism of methanesulfonic acid involves a multicomponent monooxygenase enzyme. *Microbiology* **142**, 251–260 (1996).
15. Hahnke, S. *et al.* Physiological diversity of *Roseobacter* clade bacteria co-occurring during a phytoplankton bloom in the North Sea. *Syst. Appl. Microbiol.* **36**, 39–48 (2013).
16. Geng, H. & Belas, R. Molecular mechanisms underlying *Roseobacter*–phytoplankton symbioses. *Curr. Opin. Biotechnol.* **21**, 332–338 (2010).
17. Kasalický, V. *et al.* Aerobic anoxygenic photosynthesis is commonly present within the

genus *Limnohabitans*. *Appl. Environ. Microbiol.* **84**, e02116-17 (2018).

18. Avcı, B. *et al.* Genomic and physiological analyses of '*Reinekea forsetii*' reveal a versatile opportunistic lifestyle during spring algae blooms. *Environ. Microbiol.* **19**, 1209–1221 (2017).
19. Jimenez-Infante, F. *et al.* Comprehensive genomic analyses of the OM43 clade, including a novel species from the Red Sea, indicate ecotype differentiation among marine methylotrophs. *Appl. Environ. Microbiol.* **82**, 1215–1226 (2016).
20. Giovannoni, S. J. *et al.* The small genome of an abundant coastal ocean methylotroph. *Environ. Microbiol.* **10**, 1771–1782 (2008).
21. Mincer, T. J. & Aicher, A. C. Methanol production by a broad phylogenetic array of marine phytoplankton. *PLOS ONE* **11**, e0150820 (2016).
22. Kasai, Y., Kishira, H. & Harayama, S. Bacteria belonging to the genus *Cycloclasticus* play a primary role in the degradation of aromatic hydrocarbons released in a marine environment. *Appl. Environ. Microbiol.* **68**, 5625–5633 (2002).
23. Kasai, Y., Shindo, K., Harayama, S. & Misawa, N. Molecular characterization and substrate preference of a polycyclic aromatic hydrocarbon dioxygenase from *Cycloclasticus* sp. strain A5. *Appl. Environ. Microbiol.* **69**, 6688–6697 (2003).
24. Teira, E. *et al.* Dynamics of the hydrocarbon-degrading *Cycloclasticus* bacteria during mesocosm-simulated oil spills. *Environ. Microbiol.* **9**, 2551–2562 (2007).
25. Dyksterhouse, S. E., Gray, J. P., Herwig, R. P., Lara, J. C. & Staley, J. T. *Cycloclasticus pugetii* gen. nov., sp. nov., an aromatic hydrocarbon-degrading bacterium from marine sediments. *Int. J. Syst. Bacteriol.* **45**, 116–123 (1995).
26. Sluis, M. K. *et al.* Biochemical, molecular, and genetic analyses of the acetone carboxylases from *Xanthobacter autotrophicus* strain Py2 and *Rhodobacter capsulatus* strain B10. *J. Bacteriol.* **184**, 2969–2977 (2002).
